# Tough Talks Virtual Simulation HIV Disclosure Intervention for Young Men Who Have Sex With Men: Development and Usability Testing

**DOI:** 10.2196/38354

**Published:** 2022-09-08

**Authors:** Lisa B Hightow-Weidman, Kathryn Muessig, Zach Soberano, Matthew T Rosso, Andrew Currie, Margo Adams Larsen, Kelly Knudtson, Alyssa Vecchio

**Affiliations:** 1 Institute for Global Health and Infectious Diseases University of North Carolina at Chapel Hill Chapel Hill, NC United States; 2 Virtually Better Inc Decatur, GA United States; 3 School of Medicine University of Washington Seattle, WA United States; 4 Department of Family & Community Medicine University of New Mexico Albuquerque, NM United States

**Keywords:** HIV, virtual reality, status disclosure, prevention, young men who have sex with men, artificial intelligence, medication adherence, transmission, viral load, men, sex, development, usability, testing, virtual simulation, young men, United States, behavior, social determinants

## Abstract

**Background:**

HIV status disclosure is an important decision with barriers specific to young men who have sex with men (YMSM), who have the highest rates of new HIV infections in the United States. Behavioral and social determinants of the difficulty to disclose can include fear of rejection, stigma, loss of financial stability, and lack of communication skills. Once able to disclose, a person may have increased access to social support and improved informed risk reduction conversations and medication adherence. Despite the known challenges and advantages of disclosure, there are few effective tools supporting this behavior.

**Objective:**

To address this gap in disclosure interventions, the Tough Talks (TT) app, an mHealth intervention using artificial intelligence (AI)–facilitated role-playing scenarios, was developed for YMSM. This paper reports stages of development of the integrated app and results of the usability testing.

**Methods:**

Building on the successful development and testing of a stand-alone interactive dialogue feature in phases 1-3, we conducted additional formative research to further refine and enhance the disclosure scenarios and develop and situate them within the context of a comprehensive intervention app to support disclosure. We assessed the new iteration for acceptability and relevance in a usability study with 8 YMSM with HIV. Participants completed a presurvey, app modules, and a semistructured qualitative interview.

**Results:**

TT content and activities were based on social cognitive theory and disclosure process model framework and expanded to a 4-module curriculum. The AI-facilitated scenarios used dialogue from an utterance database developed using language crowdsourced through a comic book contest. In usability testing, YMSM reported high satisfaction with TT, with 98% (31/33) of activities receiving positive ratings. Participants found the AI-facilitated scenarios and activities to be representative and relevant to their lived experiences, although they noted difficulty having nuanced disclosure conversations with the AI.

**Conclusions:**

TT was an engaging and practical intervention for self-disclosure among YMSM with HIV. Facilitating informed disclosure decisions has the potential to impact engagement in sexual risk behaviors and HIV care. More information is needed about the ideal environment, technical assistance, and clinical support for an mHealth disclosure intervention. TT is being tested as a scalable intervention in a multisite randomized controlled trial to address outstanding questions on accessibility and effect on viral suppression.

**Trial Registration:**

ClinicalTrials.gov NCT03414372; https://clinicaltrials.gov/ct2/show/NCT03414372

## Introduction

HIV status disclosure is an important and often challenging personal decision for youth with HIV. Self-disclosure, or the sharing of personal information such as one’s HIV status, with others can be an integral part of social interaction [1]. Barriers to disclosure for youth with HIV include fear of rejection, stigma, and loss of financial stability [2,3]. Moreover, youth with HIV may lack the skills needed to effectively communicate with others regarding their status [2,3]. Studies conducted with men who have sex with men (MSM) in the United States have shown that 30% to 40% of persons living with HIV inconsistently disclose their status to sex partners, with higher odds of condomless anal intercourse (CAI) and increased number of partners among those who do not disclose [4-6]. Compelling disclosure-support interventions for youth with HIV should be grounded in their experiences of disclosure and address the barriers they face.

Status disclosure among youth with HIV is influenced by multiple factors, including the relationship to the person to whom they disclose, length of time since receiving an HIV diagnosis, and means of HIV acquisition (eg, behavioral vs perinatal transmission) [2,3]. Youth with HIV who disclose are more likely to engage in safer sex practices and report better social support [7,8]. Further, disclosure has been shown to impact subsequent mental health benefits and may improve engagement in HIV care and medication adherence [2,9]. Given the low rate of antiretroviral therapy (ART) adherence among youth with HIV contrasted with the benefits of consistent viral suppression including reduced rate of transmission to sex partners, HIV status disclosure may play an integral role in ongoing efforts to end the HIV epidemic [10-12].

With new HIV infections in the United States disproportionately affecting young MSM (YMSM), it is paramount that efforts to improve disclosure self-efficacy prioritize this at-risk group [13-15]. Despite the public health and personal benefits of disclosure, there is currently a lack of effective interventions; a 2019 systematic review identified 1455 publications with disclosure interventions, 8 that met the inclusion criteria, of which only 5 were efficacious in promoting disclosure to sex partners [16].

Tough Talks (TT), a mobile health (mHealth) app-based intervention using artificial intelligence (AI)-facilitated role-playing scenarios for YMSM with HIV, was developed in 2019 to meet this need. The content developed for TT is based on social cognitive theory (SCT), and the formative research for the initial prototype has been previously described [17,18]. In summary, the formative research consisted of the first 3 phases of development that produced and tested 3 AI-facilitated role-playing scenarios for YMSM to practice HIV status disclosure [17]. Pilot testing with 11 youth with HIV showed high acceptability for the scenarios; however, user feedback indicated the need for additional disclosure-focused content to support the AI-facilitated role-plays [17].

Here we describe the final phases of development and testing to create the fully developed, multicomponent TT. This includes the participant-recommended additional content, namely the SCT-based modular curriculum and crowdsourced expansion of disclosure scenarios, along with a paradata (ie, user intervention analytics) [18,19] reporting system that tracks user activities completed, number of log-ins, time spent, etc, and an administrator portal to move TT toward final clinical testing.

## Methods

### Overall

TT was created through a collaboration between the Behavior and Technology Lab (BATLab) at the University of North Carolina at Chapel Hill (UNC-CH) and Virtually Better, Inc. (VBI). The interdisciplinary group drew upon expertise in a spectrum of fields from HIV prevention and clinical psychology to computer science and product development. Each stage of the formative work included feedback from YMSM to ensure the content, context, and function of the app was relevant and acceptable.

**Figure 1:**
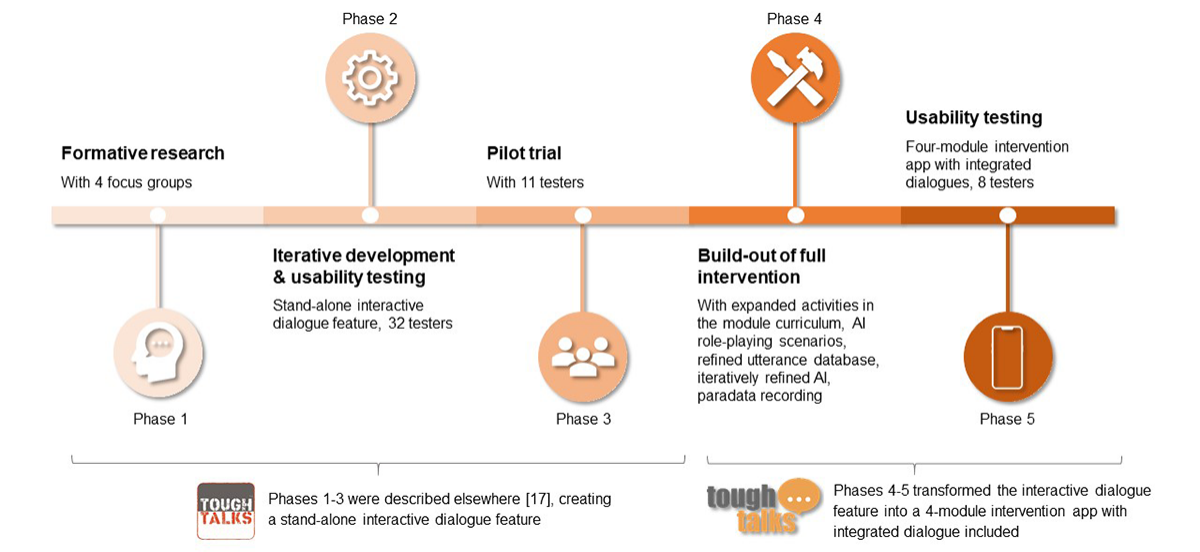
Tough Talks timeline: development of Tough Talks app, phases 1-5.

The process and results of designing this integrated, theory-based intervention are described by phases (Figure 1). Since the outcomes of each phase influence the development and methods of subsequent phases, an integrated description is presented. A stand-alone interactive dialogue feature was created and refined in phases 1 to 3 as previously presented [17]. Formative research in phase 1 began with participants from the 4 focus groups (n=58) describing past disclosure experiences, discussing disclosure strategies (barriers and facilitators) and working in pairs to develop real-life disclosure scenario conversations, and assisting in creating a stand-alone interactive dialogue feature. In phase 2, the dialogue feature was further refined through 3 rounds of usability testing with YMSM with HIV (n=32), and in phase 3, the feature was tested for preliminary efficacy with 11 new YMSM with HIV. The TT prototype at the end of phase 3 included an SCT-informed virtual dialogue feature to guide YMSM through HIV status disclosure conversations. This paper describes methods to supplement the dialogue feature with activities and educational content in response to participant feedback from earlier phases and align more closely with the SCT framework [20]. Specifically, development of the fully integrated app-based intervention and usability testing are described in phases 4 and 5, respectively.

### Phase 4 Activities

Additional AI-facilitated role-playing scenarios were developed to include more complex disclosure conversations and allow users to practice disclosing to a simulated sex partner. Authentic and realistic disclosure dialogues for these activities were sought using online crowdsourcing in the form of a comic book contest [21]. The online submission platform included 8 comic book panels that included dialogue prompts to initiate a disclosure dialogue. Panels with no introductory text were also included to encourage original submissions. Text from the situations in these panels are included in Table 1. The online comic book contest was conducted between November 2017 to February 2018 and advertised on social media (eg, Facebook, Grindr). The website provided specific submission instructions, including entry eligibility (eg, must include intelligible dialogue) and a brief tutorial on the submission process. Multiple entries were allowed and encouraged. Eligible participants were aged 16 to 29 years, born male and identify as male, and having had anal sex with another man or intended to in last 12 months. Submissions were judged by the BATLab study team and members of the youth advisory board on a scale of 1 to 5 (5 being the best) on the content of the conversations within the following 3 domains: relatability, uniqueness, and relevancy of the dialogue for the final TT disclosure scenarios.

**Table 1:** Online comic book contest disclosure scenario prompts.

Scenario	Introduction text to the disclosure situation
WTF^a^ text	You’ve hooked up with this guy a couple times, usually when you see him out. It’s very casual. You receive a text from him. “Hey. My friend just told me you’re poz. WTF. Why didn’t you tell me???”
Catching feelings	You’re meeting up with a partner you have been dating for a few weeks. You two have not yet had sex, but you both want to. You really like him but are nervous that he won’t want to continue with you if he knows you are HIV positive.
Snooping	You’re in bed cuddling with a guy you just hooked up with. He gets up to go the bathroom and walks out holding a bottle of your HIV meds.
FWB^b^ to Bae?	You’ve been hooking up with a partner randomly for about 2 months. You both start developing feelings for each other and hanging out more. You haven’t talked about each other’s status but you want to.
Protected?	You meet up with a partner you have been dating for a few weeks. You had protected sex with them a few nights ago. You two have not talked about status.
Club hook-up	You’re making out with a guy in the club and he pulls you toward the bathroom.
But I don’t like condoms	You’re about to hook up with a partner you just met after going back to his place. You realize you don’t have a condom and ask him for one. He says, “I don’t like condoms.”
Slide into those DMs^c^	You met a guy on Instagram and have been texting for about a month. It’s always really flirty, and you’ve started to really like him. You guys haven’t talked about HIV status.

^a^WTF: What the fuck.

^b^FWB: friends with benefits.

^c^DM: Direct Message.

### Ethics Approval

This study was approved (14-0345) by the institutional review board at UNC-CH.

## Results

### Overall

Module content and activities (Table 2) are grounded in the constructs of SCT and informed by the results of phases 1 to 3 [17]. Content addresses internalized HIV stigma, fear of disclosing one’s status, fear of rejection, feelings of being overwhelmed, pressure to educate others about HIV, and past negative experiences with disclosure. Cognitive factors, specifically knowledge, expectations, and attitudes surrounding disclosure, are explored through videos, educational activities, and quizzes. We also added disclosure legal requirements by state, easy to understand information about HIV, tips for how to talk to others about HIV, and safer sex facts. Environmental factors (ie, intersectional stigma, regional context, social norms) are addressed through play-through choose-your-own-adventure style narratives, and activities focused on disclosure context and timing. Behavioral factors to increase disclosure skills and self-efficacy are addressed with activities that include decisional balances around disclosure costs and benefits, sexual communication tips, and coping mechanisms for disclosing including through emotional regulation. These activities aligned with formative data in which participants described weighing the costs and benefits of disclosing their HIV status to intimate partners, friends, and family. For some, the social support made it worth it, while others learned how to accept the range the outcomes including those from people who were not as open or informed.

**Table 2:** Social cognitive theory–informed activities included in modules.

	Cognitive factors				Environmental factors				Behavioral factors		
	Knowledge	Expectations	Attitudes	Social norms		Access in community	Influence on others	Skills		Practice	Self-efficacy
0.1. Introduction and goals		✓									✓
1.0. CYOA^a^: It’s like coming out...again		✓	✓	✓						✓	✓
1.1. Disclosure and state laws	✓			✓							
1.2. What is disclosure?	✓	✓									
1.3. Who needs to know?	✓	✓									
1.4. I am (blank)		✓	✓								
1.5. What would you do?		✓	✓	✓			✓	✓		✓	
1.6. Virtual disclosure practice		✓	✓			✓		✓		✓	✓
2.0. CYOA: The dating game...with a twist		✓	✓			✓				✓	✓
2.1. Your past experiences		✓	✓								
2.2. To disclose or not to disclose?		✓	✓				✓	✓		✓	
2.3. Is now the time to do it?	✓	✓		✓							
2.4. Right time, right place	✓	✓	✓				✓	✓		✓	
3.0. CYOA: He likes me...he likes me not		✓	✓	✓						✓	✓
3.1. Breaking the ice (subtly)	✓	✓	✓				✓	✓		✓	
3.2. Tell it over text	✓	✓	✓	✓			✓	✓			
3.3. Conversation starters	✓	✓	✓					✓		✓	
3.4. The cat’s out of the bag	✓	✓	✓					✓		✓	
4.0. CYOA: How did you find out?		✓	✓	✓						✓	✓
4.1. Q&A^b^: HIV edition	✓	✓						✓		✓	
4.2. What are you willing to answer?	✓	✓	✓				✓	✓		✓	✓
4.3. How would you answer?	✓	✓	✓	✓				✓		✓	
4.4. What am I most afraid of?	✓	✓	✓	✓							
4.5. Get out while you can	✓	✓	✓				✓	✓		✓	
4.7. Reflections 2		✓	✓								✓

^a^CYOA: choose your own adventure.

^b^Q&A: questions and answers.

### Expanded AI Scenarios: Comic Book Contest and Refinement of Utterance Database

**Figure 2:**
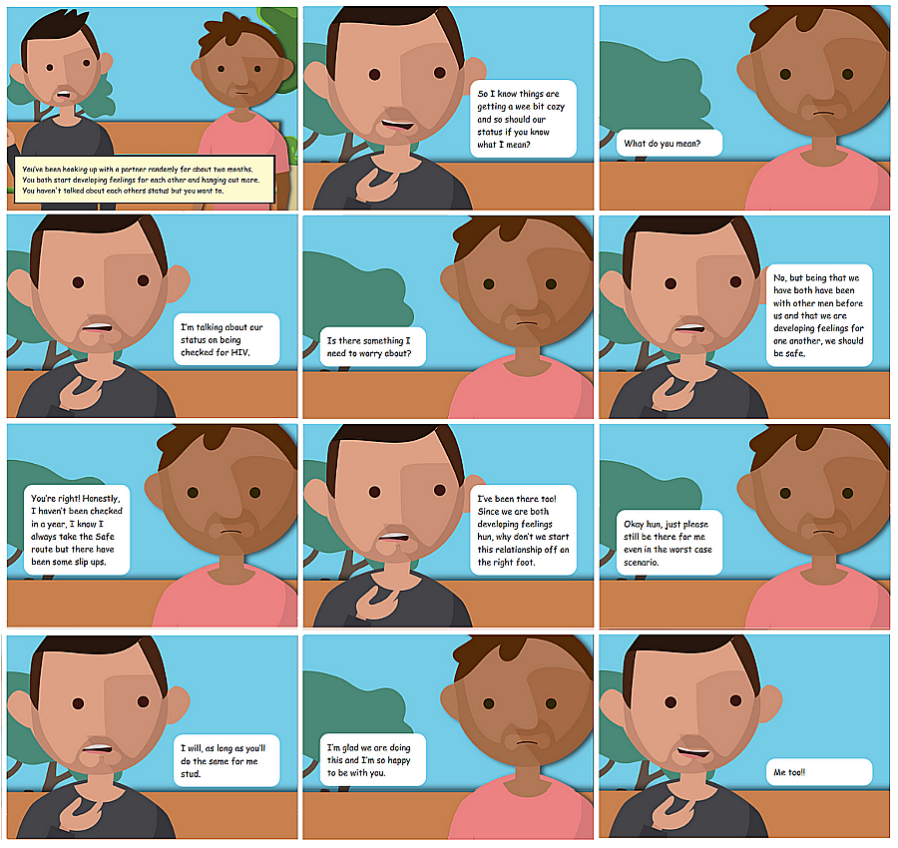
Online comic book contest winning disclosure dialogue.

A total of 76 eligible and consented participants created accounts on the contest website; 21 completed comics were submitted during the contest, including 16 daily winners, 4 weekly winners, and 1 grand prize winner (Figure 2). We incorporated the comics’ relevant context, situations, and language into the module activities (eg, “Tell it over text”) and the AI-facilitated role-playing disclosure scenarios. This created a richer selection of responses for the utterance database (“Honestly, I haven’t been checked in a year. I know I always take the safe route, but there have been some slip ups” as a response to a TT user’s question about HIV status or testing), the collection of responses the AI system could use as the virtual character based on user conversation. The responses identified as reactions to disclosure were classified as being positive, negative, or neutral so the virtual character (ie, avatar) could be programmed and properly respond with a full range of reactions to the app user’s status disclosure.

### Development of an Integrated App-Based Intervention

The final intervention consists of 4 modules containing 24 activities and 8 AI-facilitated role-playing scenarios. Each module builds on the previous one in terms of disclosure knowledge, decision-making, and practice. All modules (Understanding disclosure, Should I disclose?, How do I disclose?, Preparing for the outcome) begin with a choose-your-own-adventure style game featuring a young man living with HIV navigating how to disclose to family, friends, and romantic and sexual partners (Table 1). Modules also include 4 to 5 informational/skill-building activities, goal setting, and reflection activities. Modules also include an embedded AI-facilitated role-playing scenario with 2 variable partner reaction outcomes (positive, neutral, negative) (Table 3). Positive reactions were those where the avatar was explicitly supportive, neutral reactions were those in which the avatar was neither explicitly supportive nor unsupportive, and negative reactions were those in which the avatar was explicitly unsupportive.

**Table 3:** Artificial intelligence–facilitated role-play scenarios and partner reaction outcomes.

Module	Scenario	Have they previously had sex?	First reaction outcome	Second reaction outcome
1	You met a guy on Instagram and have been texting for about a month. It’s always really flirty, and you’ve started to really like him. You guys haven’t talked about HIV status.	No	Positive	Neutral
2	You’re about to hook up with a partner you just met after going back to his place. You realize you don’t have a condom and ask him for one. He says, “I don’t like condoms.”	No	Neutral	Negative
3	You meet up with a partner that you have been dating for a few weeks. You had protected anal sex with him using condoms a few nights ago. You two have not talked about status.	Yes	Negative	Positive
4	You’ve been hooking up with a partner randomly for about 2 months. You have had condomless sex. You both start developing feelings for each other and hanging out more. You haven’t talked about each other’s status, but you want to.	Yes, condomless	Positive (on PrEP^a^)	Neutral

^a^PrEP: preexposure prophylaxis.

### App Backend Database: Enhancing the Avatar Responses and Capturing Intervention Paradata

The SCT-based activities and disclosure scenarios collected from the comic book contest were integrated into TT by VBI. Members of the research team completed internal usability testing of TT, with particular attention to coding the AI to properly select an appropriate avatar response based on the conversation’s tone and context. In the fully automated system, the natural language processing system [17] chooses a response it determines fits best for the simulated sex partner based on the scenario and TT user input. To address limitations to the AI program we identified such as its ability to understand complex sentences from the TT user, we developed and tested a semiautomated, “wizard-of-oz”–driven system in which the AI program provided suggested responses to a staff member through the administrator portal but allowed the staff member to either accept this suggestion or use another one to respond as the simulated character to the TT user. Of note, all the possible responses in the semiautomated version were the same as those contained in the automated version (eg, the staff member could not craft their own response). VBI created a backend database to track intervention paradata consisting of all participant activity conducted within the app (eg, length of time app was used, modules and activities completed, disclosure scenario chat logs). For the study team, VBI created an administrator portal for TT where the research administrator could view the coded user ID, date/time of last activity, and the cumulative number of activities completed. This backend feature captures the intervention paradata and allows research administrators to monitor TT use.

### Usability Testing of Fully Developed Intervention

Once the prototype was fully developed, a usability assessment of TT was conducted in North Carolina from July to October 2018. Eligibility criteria included being born male and identifying as male, being aged 16 to 29 years, self-reporting living with HIV, and being fluent in English, and having had at least one episode of CAI with another man or transgender woman in the past 6 months. Recruitment was primarily through flyers and referrals from local HIV clinics.

Study team members conducted usability sessions in private rooms at community- and clinic-based study sites. Each session lasted 60 to 90 minutes. Participants completed a presurvey (Multimedia Appendix 1), reviewed TT modules, and participated in semistructured qualitative interviews. The presurvey included questions on demographics, disclosure experience (ever disclosed to a sexual partner, anal or vaginal sex without disclosure in past 12 months), use of technology, and experience with AI-facilitated games/interventions (“Do you have any AI-related experience? This can be through online virtual worlds, games, etc.”). Participants each reviewed 2 of the 4 modules. Participants were given a workbook that included 2 questions about every activity (liked activity and activity length) as well as a notes section for every activity.

Following the administration of the modules, participants completed a semistructured interview to rate TT on visual appeal, acceptability, ease of use, and user experience. In these interviews, participants were asked to assess the relevance and accuracy of the conversations as compared to their prior disclosure experiences (or anticipated experiences), emotional impact of using the program, how and where they could see themselves using the program, perceived usefulness of the program, and suggestions for improvements. Usability interview sessions were audiorecorded. Participants received $50 for participation. Results of the survey and quantitative workbook responses were reviewed and analyzed in Excel (Microsoft Corp) for demographics and responses held by a majority of the participants. Thematic analysis of usability qualitative interview was conducted through multiple phases of reading and coding by study team members experienced in public health qualitative data analysis. Given the size of the cohort, qualitative data analysis software was not used for coding; we opted instead for a collaborative process of extracting the themes from interviews and workbooks.

### Participant Characteristics

A total of 8 YMSM living with HIV were enrolled (Table 4); mean age was 27.6 years, and all identified as Black. Mean time since HIV diagnosis was 4.6 years (range 5 months to 12 years). All participants were in-care, virally suppressed, and self-reported ≥90% ART adherence in the last month. All reported anal sex in the last 3 months, 88% (7/8) of the YMSM reported CAI, and half (4/8, 50%) reported an STI in the last 6 months. Of the YMSM, 62% (6/8) believed people are rejected when they disclose; 88% (7/8) reported disclosing their status to a sex partner. Among those reporting CAI, 57% (4/8) of the YMSM disclosed to some partners, 29% (2/8) disclosed to all partners, and 14% (1/8) did not disclose to partners.

**Table 4:** Participant characteristics (n=8).

			Value
Age (years), mean			27.6
**Race/ethnicity, n (%)**			
	Black or African American, non-Hispanic	8 (100)	
**Time since HIV diagnosis (years)**			
	Average	4.6	
	Minimum	0.4	
	Maximum	12	
Health care, n (%)			8 (100)
Virally suppressed, n (%)			8 (100)
Self-reported ART^a^ adherence ≥90%, n (%)			8 (100)
**Anal intercourse, n (%)**			
	Past 3 months	8 (100)	
	Condomless anal intercourse	7 (88)	
STI^b^ in the last 6 months			4 (50)
Rejected/stigma with disclosure, n (%)			5 (63)
Disclosure to sex partner, n (%)			7 (88)
**Disclosure among CAI^c^ (n=7), n (%)**			
	Disclosed to some partners	4 (57)	
	Disclosed to all partners	2 (29)	
	Did not disclose to partners	1 (14)	

^a^ART: antiretroviral therapy.

^b^STI: sexually transmitted infection.

^c^CAI: condomless anal intercourse.

### Usability Feedback

All participants reported high satisfaction with TT, with 93% (31/33) of activities rated positively. There was general ease with using TT and learning new information. The most common feedback on individual activities centered on activity length, specifically that the activities were too long. Participants reported that information provided in TT was useful and new to them. They requested more activities that incorporated information on disclosing to family, dating, and additional information pertaining to HIV and state disclosure laws.

Participants offered feedback on specific components of the intervention. They particularly enjoyed the choose-your-own-adventure style games since they related to the character’s journey. Many mentioned that they had similar situations with their siblings or could see themselves having those conversations with friends. Many noted that they would repeat those activities if they had the chance. There were positive comments regarding the overall design and graphics.

Participants had mixed reactions and opinions about the AI-facilitated role-playing scenarios. While most participants found them to be the most interesting part of the app, specifically citing that they were “useful and realistic,” 3 YMSM reported that receiving negative responses from the avatar could be emotionally difficult at times. One participant specifically commented that the intervention should provide more emotional support after the simulated sex partner reacts negatively to their disclosure. Two participants noted the AI-facilitated role-playing scenarios had noticeable bugs (eg, slowness or confusing simulated sex partner responses) but still found the activity useful.

The usability study participants had to type out the conversation with the simulated sex partner as opposed to being able to speak out loud. Many participants noted that this, among other factors, took away from the realistic nature of the disclosure conversation practice.

One participant appreciated the overall tone of TT and how it focused on giving back their identity and “reinforces being yourself and that HIV doesn’t affect you.” Many participants noted that they would have found TT useful when they were first diagnosed instead of having to build their own skills to learn to disclose their status. They explained the challenges of disclosing to family, peers, and partners. One participant described the constant stress, even if at a low level, of not disclosing. Some of the fears of disclosure included partner rejection, family judgement, and physical violence.

### Implications of the Usability Study to Further Intervention Design

Changes to the design and content of the app based on participant feedback were implemented before launching the randomized controlled trial (RCT).

Specific changes are noted in Table 5. We updated the titles and order of the activities and modules to make the sections feel less school-like or childish. Features, including buttons for forgotten passwords and returning to the home screen, were modified to make TT more user-friendly. Many activities were reduced in length (eg, from around 5 minutes to 2-3 minutes) based on participant feedback. The final time for each module was reduced from 60 minutes to 45 minutes, and the overall TT intervention was shortened from 4 hours to 3 hours.

**Figure 3:**
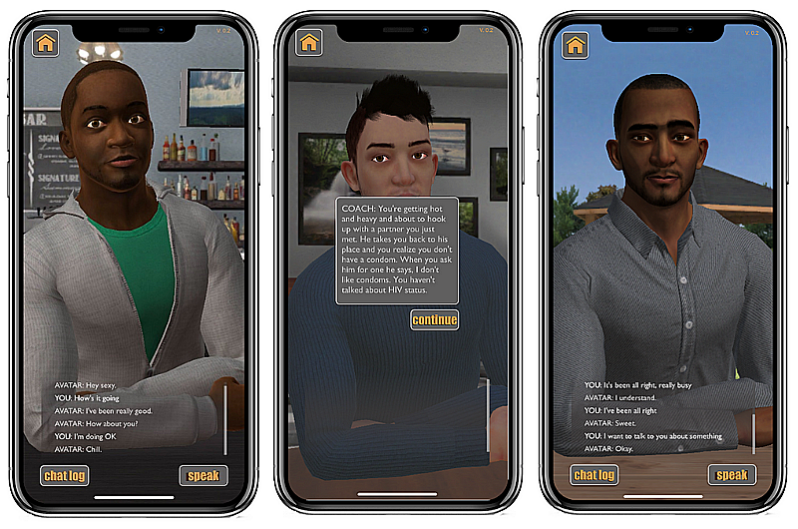
Screen shots of artificial intelligence–facilitated role plays.

The most significant modifications proposed were to the AI technology. Speech recognition was added freeing participants from having to type responses. A chat log history box was added to the bottom of the screen to allow the user to review the disclosure discussion (Figure 3). Changes were made to address emotional concerns: (1) coach dialogue was enhanced to help guide users through the scenario; (2) reflection activities were added to postdisclosure scenarios to help users process information received during the scenario, and (3) access to virtual and in-person support as needed, including local and online resources for sexual and mental health and staff and clinic contact information, was confirmed.

**Table 5:** Changes to activities based on user feedback.

Module and activity			Title		Activity	Integrated feedback
**Introduction**						
	0	Your coach		Meet the coach		
	0.1	Welcome!		Coach explains what to expect in app		
	0.2	Let’s set some goals!		Set both short-term and long-term goals		Split goals into separate sections
**Module 1: Understanding disclosure**						
	1	It’s like coming out... again		CYOA^a^ deciding if/how to disclose to a sister		Added animation to text boxes
	1.1	What is disclosure?		Animated video explaining disclosure		Split into separate shorter videos
	1.2	Disclosure and state laws		Differences in state laws about disclosure		Updated to include all 50 states
	1.3	Who needs to know?		Quiz about who people need to disclose to		
	1.4	I am (blank)...		Fill-in-the-blank about role of HIV stigma		Updated to be more age appropriate
	1.5	What would you do?		Give advice to a friend about disclosure		Improved texting simulation
	1.6	Virtual disclosure practice		Role-play status disclosure to a virtual partner		
**Module 2: Should I disclose?**						
	2	The dating game...with a twist		CYOA about disclosing status on dating apps		Added animation to text boxes
	2.1	Your past experiences		Reflect on previous disclosure experiences		Added colors to improve user experience
	2.2	To disclose or not disclose?		Sort positive and negative outcomes of disclosure		Shortened activity and clarified language
	2.3	Is now the time to do it?		Animated video about disclosure circumstances		Video added from previous section
	2.4	Right time, right place		Pros and cons of different settings to disclose		
	2.5	Virtual disclosure practice		Role-play status disclosure to a virtual partner		
	2.6	Reflection		First impressions and revisit initial goals		
**Module 3: How do I disclose?**						
	3	He likes me...he likes me not		CYOA about dating a coworker		Added animation to text boxes
	3.1	Breaking the ice subtly		Animated video with tips for subtle disclosure		
	3.2	Tell it over text		Examples of disclosure conversations via text		Improved texting simulation
	3.3	Conversation starters—bracket challenge		Choose favorite disclosure conversation starters		Improved visual user experience
	3.4	The cat’s out of the bag		Consider how to respond to person finding out status		Added text entry box to be interactive
	3.5	Virtual disclosure practice		Role-play status disclosure to a virtual partner		
**Module 4: Preparing for the outcome**						
	4	How did you find out?		CYOA when a friend inadvertently finds out		Added animation to text boxes
	4.1	Q&A^b^: HIV edition		Answers to FAQs^c^ about HIV postdisclosure		Improved visual user experience
	4.2	What are you willing to answer?		Self-reflection on what you’ll personally answer		Added “other” text entry option
	4.3	How would you answer?		Fill-in-the-blank comic-style conversation activity		
	4.4	What am I most afraid of?		Recognize fears surrounding disclosure		
	4.5	Get out while you can		Tips if disclosure goes badly		
	4.6	Virtual disclosure practice		Role-play status disclosure to a virtual partner		
	4.7	Reflection		Final check-in and revisit goals		

^a^CYOA: choose your own adventure.

^b^Q&A: questions and answers

^c^FAQ: frequently asked questions.

## Discussion

### Principal Findings

TT, a behavioral intervention to practice HIV status disclosure, was developed over multiple phases of research and participant testing. Building on the successful development and testing of a virtual simulation prototype that demonstrated AI-driven scenarios were both feasible and acceptable to YMSM to practice HIV status disclosure with sex partners (phases 1 to 3) [17], we transformed the virtual prototype into a modular disclosure curriculum contained within an interventional mobile health app tested with 8 YMSM living with HIV (phases 4 and 5). Throughout the development process, the perspectives of YMSM were used to create a relevant and engaging intervention. Usability testing found that the intervention was both feasible and acceptable among members of the priority population, who reported learning new information after engaging in the TT activities.

Participants in the early phases of the intervention development confirmed the barriers and potential benefits of HIV status disclosure that have been discussed in previous literature [22,23]. However, they also identified personal priorities for disclosure decisions and outcomes specific to their age, race, relationship status, time since HIV diagnosis, and region. Participant feedback directed the development of the 4-module curriculum that includes general HIV information and communication skill-building activities. The main emphasis throughout the modular curriculum is practicing disclosure decisions, which gives users the ability to think through possible scenarios and create a personal strategy to approach disclosure opportunities. This is intended to reduce the uncertainty and anxiety experienced prior to disclosing one’s status. The RCT will determine whether it is more helpful to complete these modules at the user’s own pace or in an environment where they can have technical or emotional support with the app. It will also address the timing of intervention, including whether the app is particularly useful for those recently diagnosed with HIV. This will help to guide the implementation of the app in the clinical setting in terms of timing and staffing.

AI has been successfully implemented in behavioral interventions for other chronic diseases [24,25], and the initial phases of TT confirmed its effectiveness for YMSM with HIV [17]. However, the emotional and psychological complexity of status disclosure requires additional development of the automated, interactive system used in TT. The crowdsourced disclosure scenarios from the comic book contest supplied additional content to the utterance database. This training data from the target population will enhance the simulated sex partner’s dialogue capacity. The RCT will include a comparison of the acceptability and effectiveness of the AI (fully automated)– and wizard-of-oz (semiautomated)–supported dialogue platform.

### Limitations

Although participants reported that the virtual scenarios were reminiscent of their own disclosure experiences, texting input took away from the realistic nature of the conversation. Thus, the app was modified to enable the simulated sex partners to respond to spoken disclosure. Virtual reality interventions have been used for treating anxiety and other psychological conditions [26], and previous studies have shown the emotional release of disclosing [27]. Subanalyses in the RCT will assess the potential therapeutic benefit of stating one’s HIV status aloud. There were limitations to the comic book consent and usability study. The crowdsourcing method for the comic book allowed our team to reach a wider audience, but the participants did not indicate their HIV status. The usability study had a small sample from one geographic location, and all identified as Black or African American, which limits the generalizability of the findings. We plan to enroll a more diverse cohort for the RCT that includes a large proportion of people of color since HIV disproportionally affects populations with systemic health disparities. The usability study participants were recruited from a clinical setting, so many already had established care. These factors were taken into consideration in designing eligibility criteria for the RCT, which is the next phase for testing TT. The limitations to the use of the paradata will be noted during the RCT.

### Conclusion

Disclosure is not a one-time event, but a complex series of personal decisions and actions that will play out over the lifetime of youth with HIV. Despite advances in HIV care, HIV disclosure interventions that provide skills and information for YMSM living with HIV to make their own disclosure decisions are still needed regardless of time since diagnosis, viral suppression, or past disclosure experiences. TT was found to be an engaging and practical intervention for self-disclosure among YMSM living with HIV. Facilitating informed disclosure decisions has the potential to impact engagement in sexual risk behaviors and ART adherence. More information is needed about the ideal environment (eg, at home, in clinic) and technical and staff support required for implementation of an mHealth disclosure intervention. To that end, TT will be tested for effectiveness in a multisite RCT.

## Appendix

Multimedia Appendix 1 Tough Talks technical pilot presurvey.

## Abbreviations

AI: artificial intelligence
ART: antiretroviral therapy
BATLab: Behavior and Technology Lab
CAI: condomless anal intercourse
mHealth: mobile health
MSM: men who have sex with men
RCT: randomized control trial
SCT: social cognitive theory
TT: Tough Talks
UNC-CH: University of North Carolina at Chapel Hill
VBI: Virtually Better, Inc
YMSM: young MSM
