# Supplementary material for: Tough Talks Virtual Simulation HIV Disclosure Intervention for Young Men Who Have Sex With Men: Development and Usability Testing
Source: JMIR Form Res. 2022 Sep 8;6(9):e38354. doi: 10.2196/38354 (PMC9501675; doi:10.2196/38354)
Supplement: Multimedia Appendix 1 [file formative_v6i9e38354_app1.docx]

Multimedia Appendix 1. Tough Talks Technical Pilot Presurvey

Start of Block: Default Question Block

Q0 Thank you for participating in the Tough Talks II technical pilot! The goal of Tough Talks II is to create a virtual-reality based intervention program that uses artificial intelligence and virtual support to help young, HIV positive men navigate through difficult conversations – specifically disclosing their status to their sexual partners.  

The purpose of our research study is to get opinions and feedback on our app and its usability. Try to answer every question as best you can. If you need help, please ask the study staff. Your answers will be used only for research purposes. Let's get started!

Domain 1 Demographics

| 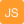 |
| --- |

Q1 Select a Date:


(Note, this question is intelligent about month lengths and leap years. You can set the year range by editing the first lines in the JS editor)

|  | Month | Year |
| --- | --- | --- |
|  |  |  |
| Please Select: (1) | ▼ January (1 ... December (12) | ▼ 1900 (1 ... 2049 (150) |

Q1 What is your date of birth?

|  | Month | Year |
| --- | --- | --- |
|  |  |  |
| Click to write Statement 1 (4) | ▼ Answer 1 (1 ... Answer 2 (2) | ▼ Answer 1 (1 ... Answer 2 (2) |

Q2 Do you consider yourself Hispanic or Latino?

- Yes (1)
- No (2)
- Decline to answer (3)

Q3 How would you best describe your race/ethnicity? You may choose more than one.

- American Indian / Alaskan Native (1)
- Asian (2)
- Black or African American (3)
- Native Hawaiian or Other Pacific Islander (4)
- White (5)
- Other, specify (6) ________________________________________________
- Decline to answer (7)

Q4 What is your current sexual identity?

- Gay, homosexual, same gender loving (1)
- Bisexual (2)
- Queer (3)
- Straight or heterosexual (4)
- Other, specify (5) ________________________________________________
- Decline to answer (6)

Q5 How open are you about your sexual identity to your primary medical care provider? A primary medical care provider is a health care provider that you see regularly, such as a pediatrician, a doctor, or other health care provider.

- Primary medical provider DEFINITELY does NOT know your sexual identity (1)
- Primary medical provider MIGHT know about your sexual identity, but it is NEVER talked about (2)
- Primary medical provider PROBABLY knows about your sexual identity, but it is NEVER talked about (3)
- Primary medical provider PROBABLY knows about your sexual identity, but it is RARELY talked about (4)
- Primary medical provider DEFINITELY knows about your sexual identity, but it is RARELY talked about (5)
- Primary medical provider DEFINITELY knows about your sexual identity, and it is SOMETIMES talked about (6)

Q6 Are you currently enrolled in school? "School” could mean a school or program where you are working toward a high school diploma, GED, or college/technical degree.

- Yes (1)
- No (2)
- Decline to answer (3)

| Page Break |  |
| --- | --- |

Display This Question:

If Are you currently enrolled in school? "School” could mean a school or program where you are worki... = Yes

Q7 What is your current grade level in school?

- 6th-8th grade (1)
- 9th-12th grade (2)
- GED program (3)
- Technical or vocational school (4)
- Two-year college (5)
- Four-year college (6)
- Graduate school (7)
- Decline to answer (8)

| Page Break |  |
| --- | --- |

Display This Question:

If Are you currently enrolled in school? "School” could mean a school or program where you are worki... != Yes

Q8 What is the highest level of school that you have completed?

- None, no formal schooling (1)
- 6th-8th grade (2)
- 9th-12th grade (3)
- High school diploma (4)
- High school certificate of completion (no diploma) (5)
- GED (6)
- Some college, technical school, or vocational school (7)
- Technical or vocational school graduate (8)
- Two-year college graduate (9)
- Four-year college graduate (10)
- Some graduate school (11)
- Master's degree or above (12)
- Decline to answer (13)

Q9 Are you currently employed?

- Yes (1)
- No (2)
- Decline to answer (3)

| Page Break |  |
| --- | --- |

Display This Question:

If Are you currently employed? = Yes

Q10 Are you employed full-time or part-time? Part-time means that you work less than 35 hours per week during most weeks.

- Full time (1)
- Part time (2)
- Decline to answer (3)

Q11 In the past 3 months, how often did you or your family have to cut meal size or skip a meal because there was not enough money for food?

- Almost every week (1)
- Several weeks but not every week (2)
- Only a few weeks (3)
- Did not have to skip or cut the size of meals (4)
- Decline to answer (5)

Q12 In the past 30 days, have you spent at least one night (check all that apply):

- In a shelter? (1)
- In a public place not intended for sleeping (e.g., bus station, car, abandoned building)? (2)
- On the street or anywhere outside (e.g., park, sidewalk)? (3)
- Temporarily doubled up with a friend or family member? (4)
- In a temporary housing program? (5)
- In a welfare or voucher hotel/motel? (6)
- In jail, prison, or a halfway house? (7)
- In drug treatment, a detox unit, or drug program housing? (8)
- In a hospital, nursing home, or hospice? (9)
- I have not spent a night in any of the above places. (10)
- Decline to answer (11)

Q13 Have you ever been arrested?

- Yes (1)
- No (2)
- Decline to answer (3)

| Page Break |  |
| --- | --- |

Display This Question:

If Have you ever been arrested? = Yes

Q14 Have you been arrested in the past 3 months?

- Yes (1)
- No (2)
- Decline to answer (3)

Q15 Have you ever been put in jail, prison or juvenile detention (juvy)?

- Yes (1)
- No (2)
- Decline to answer (3)

| Page Break |  |
| --- | --- |

Display This Question:

If Have you ever been put in jail, prison or juvenile detention (juvy)? = Yes

Q16 Have you been put in jail, prison or juvenile detention (juvy) in the past 3 months?

- Yes (1)
- No (2)
- Decline to answer (3)

| Page Break |  |
| --- | --- |

Domain 2 Relationships

Q17 How do you define your primary relationship status?

- I am single, and having sex with others (1)
- I am single, and not having sex with others (2)
- I am casually dating (3)
- I have a boyfriend or girlfriend (4)
- I have a partner or lover (5)
- Although we lack a legal commitment, I am with a partner and we have had a commitment ceremony (6)
- I am in a civil union or domestic partnership (7)
- I am legally married (8)
- Decline to answer (9)

| Page Break |  |
| --- | --- |

Display This Question:

If How do you define your primary relationship status? = I have a boyfriend or girlfriend

Or How do you define your primary relationship status? = I have a partner or lover

Or How do you define your primary relationship status? = Although we lack a legal commitment, I am with a partner and we have had a commitment ceremony

Or How do you define your primary relationship status? = I am in a civil union or domestic partnership

Or How do you define your primary relationship status? = I am legally married

Q18 How do you and your partner handle sex outside of your relationship?

- Neither of us has sex with others; we are monogamous (1)
- Only I have sex with others (2)
- Only my partner has sex with others (3)
- Both of us have sex with others separately (4)
- Both of us have sex with others together (5)
- We both have sex with others separately and together (6)
- I have sex with others, but I don’t know about my partner (7)
- I don’t have sex with others, but I don’t know about my partner (8)
- Decline to answer (9)

Domain 3 Technology Usage

Q19 Which of the following devices do you own? (Check all that apply)

- Cell phone (basic mobile phone for calling or texting; does not have internet access, apps, or a touch screen) (1)
- Smartphone (advanced mobile phone with internet access, apps, and a touch screen) (2)
- Desktop computer (3)
- Laptop computer (4)
- Tablet computer (5)
- E-book reader (6)
- Fitness tracker or smart watch (7)
- Another device, specify (8) ________________________________________________
- Decline to answer (9)

Q20 How do you usually access the internet? (Check all that apply)

- Smartphone (1)
- Desktop computer (2)
- Laptop computer (3)
- Tablet computer (4)
- E-book reader (5)
- Another method, specify (6) ________________________________________________
- Decline to answer (7)

Display This Question:

If How do you usually access the internet? (Check all that apply) = Smartphone

Or How do you usually access the internet? (Check all that apply) = Desktop computer

Or How do you usually access the internet? (Check all that apply) = Laptop computer

Or How do you usually access the internet? (Check all that apply) = Tablet computer

Or How do you usually access the internet? (Check all that apply) = E-book reader

Or How do you usually access the internet? (Check all that apply) = Another method, specify

Carry Forward Selected Choices from "How do you usually access the internet? (Check all that apply)"

| 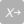 |
| --- |

Q21 How do you access the internet most frequently? (Rank your answers)

______ Smartphone (1)

______ Desktop computer (2)

______ Laptop computer (3)

______ Tablet computer (4)

______ E-book reader (5)

______ Another method, specify (6)

______ Decline to answer (7)

| Page Break |  |
| --- | --- |

Display This Question:

If Which of the following devices do you own? (Check all that apply) = Smartphone (advanced mobile phone with internet access, apps, and a touch screen)

Q22 What operating system/platform does your primary smartphone use?

- Apple (iOS) (1)
- Android (2)
- I don't know (3)
- Other, specify (4) ________________________________________________
- Decline to answer (5)

| Page Break |  |
| --- | --- |

Display This Question:

If Which of the following devices do you own? (Check all that apply) = Cell phone (basic mobile phone for calling or texting; does not have internet access, apps, or a touch screen)

Or Which of the following devices do you own? (Check all that apply) = Smartphone (advanced mobile phone with internet access, apps, and a touch screen)

Q23 What kind of Cell phone or Smartphone service do you have?

- I have a prepaid account (1)
- I have a monthly contract (2)
- I’m on a shared plan (3)
- I don’t know (4)
- None of the above (5)
- Decline to answer (6)

| Page Break |  |
| --- | --- |

Display This Question:

If Which of the following devices do you own? (Check all that apply) = Cell phone (basic mobile phone for calling or texting; does not have internet access, apps, or a touch screen)

Or Which of the following devices do you own? (Check all that apply) = Smartphone (advanced mobile phone with internet access, apps, and a touch screen)

Q24 In the past year, was your phone service ever disconnected because you could not pay the bill or because your phone was lost or stolen?

- Yes (1)
- No (2)
- Decline to answer (3)

| Page Break |  |
| --- | --- |

Display This Question:

If In the past year, was your phone service ever disconnected because you could not pay the bill or... = Yes

Q25 How many times in the last year has your phone been disconnected?

- Once (1)
- Twice (2)
- 3 to 5 times (3)
- More than 5 times (4)
- Decline to answer (5)

| Page Break |  |
| --- | --- |

Display This Question:

If In the past year, was your phone service ever disconnected because you could not pay the bill or... = Yes

Q26 The last time your phone was disconnected, for how long was it disconnected?

- 1 day or less (1)
- 2 to 7 days (2)
- 1 to 4 weeks (3)
- 1 month or more (4)
- Decline to answer (5)

Q27 Internet Time, Frequency

Q28 On average, how many hours per day do you spend on the internet (online) outside of your school or work responsibilities?

- No hours (1)
- Less than an hour (2)
- 1 to 3 hours (3)
- 4 to 6 hours (4)
- 7 to 9 hours (5)
- 10 to 12 hours (6)
- 13 to 15 hours (7)
- 16 hours or more (8)
- Decline to answer (9)

| Page Break |  |
| --- | --- |

Display This Question:

If Which of the following devices do you own? (Check all that apply) = Smartphone (advanced mobile phone with internet access, apps, and a touch screen)

Q29 How often do you use apps on your smartphone (for example: Facebook, dating apps, banking apps, Snapchat)?

- More than once a day (1)
- About once a day (2)
- A few times a week (3)
- About once a week (4)
- Less than once a week (5)
- I do not use apps on my phone (6)
- Decline to answer (7)

Q30 How often do you use websites or smartphone apps for the following reasons:

|  | Never (1) | Rarely (2) | Sometimes (3) | Often (4) | Decline to answer (5) |
| --- | --- | --- | --- | --- | --- |
| Make new friends (1) |  |  |  |  |  |
| Chat with friends (2) |  |  |  |  |  |
| Chat with family (3) |  |  |  |  |  |
| Find a date (4) |  |  |  |  |  |
| Meet partners for casual sex (5) |  |  |  |  |  |
| Look for work opportunities (6) |  |  |  |  |  |
| Track health behaviors (diet, exercise, medication management, etc.) (7) |  |  |  |  |  |
| Get information about HIV or other STIs (8) |  |  |  |  |  |
| Get other health or medical information (9) |  |  |  |  |  |
| Create event reminders (take a daily pill, exercise, etc.) (10) |  |  |  |  |  |

Q31 Please indicate your agreement with the following statements:

|  | Strongly Disagree (1) | Disagree (2) | Undecided (3) | Agree (4) | Strongly Agree (5) | Decline to answer (6) |
| --- | --- | --- | --- | --- | --- | --- |
| I know what health resources are available on the Internet. (1) |  |  |  |  |  |  |
| I know how to find helpful health resources on the Internet. (2) |  |  |  |  |  |  |
| I know how to use the Internet to answer my questions about health. (3) |  |  |  |  |  |  |
| I know how to use the health information I find on the Internet to help me. (4) |  |  |  |  |  |  |
| I have the skills I need to evaluate the health resources I find on the Internet. (5) |  |  |  |  |  |  |
| I can tell high quality health resources from low quality health resources on the Internet. (6) |  |  |  |  |  |  |
| I feel confident in using information from the Internet to make health decisions. (7) |  |  |  |  |  |  |
| I know where to find helpful health resources on the Internet. (8) |  |  |  |  |  |  |

| Page Break |  |
| --- | --- |

Q32 HIV Positive Cascade

Q33 When did you first test positive for HIV? Please provide the month and year (MMYYYY)

________________________________________________________________

Q34 Have you ever been seen by a doctor, nurse, or other health care provider for a medical evaluation or care related to your HIV infection?

- Yes (1)
- No (2)

Display This Question:

If Have you ever been seen by a doctor, nurse, or other health care provider for a medical evaluatio... = Yes

Q35 When did you first go to a health care provider after learning you had HIV? Please provide the month and year (MMYYYY)

________________________________________________________________

Display This Question:

If Have you ever been seen by a doctor, nurse, or other health care provider for a medical evaluatio... = Yes

Q36 When did you last go to a health care  provider for HIV care? Please provide the month and year (MMYYYY)

________________________________________________________________

Display This Question:

If Have you ever been seen by a doctor, nurse, or other health care provider for a medical evaluatio... = Yes

Q37 Was there a time in the past after you first engaged in HIV care that you went 6 months or longer without a medical visit with your HIV provider?

- Yes (1)
- No (2)

| 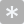 |
| --- |

Q38 In the last 12 months, how many scheduled appointments did you have? By scheduled appointments, we mean routine appointments (not walk‐in or urgent care appointments for acute/urgent issue) that were planned and booked, whether you attended them or not.

________________________________________________________________

| 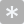 |
| --- |

Q39 In last 12 months, how many of your scheduled appointments did you miss because you didn’t show or forgot? By scheduled appointments, we mean routine appointments, not walk‐in or urgent care appointments for acute/urgent issue.

________________________________________________________________

Q40 When was the last time you missed a scheduled appointment for HIV medical care? By scheduled appointments, we mean routine appointments, not walk‐in or urgent care appointments for acute/urgent issue. Would you say:

- Within the last month (1)
- 2 months ago (2)
- 3 months ago (3)
- 4 months ago (4)
- 5 months ago (5)
- 6 months ago (6)
- More than 6 months ago (7)

Q41 Have you ever had an HIV viral load (VL) test?

- Yes (1)
- No (2)

Display This Question:

If Have you ever had an HIV viral load (VL) test? = Yes

Q42 What month and year was your most recent HIV viral load test? Please provide the month and year MMYYYY

________________________________________________________________

Display This Question:

If Have you ever had an HIV viral load (VL) test? = Yes

Q43 What was the result of your most recent viral load test? Please select one of the answers that most closely matches your last viral load test results in viral copies/ml.

- Below the level of detection, undetectable (1)
- Detectable, but less than 5,000 (2)
- 5,000 - 10,000 (3)
- Greater than 10,000 (4)
- I don't know (5)

Q44 Have you ever had a CD4 count test?

- Yes (1)
- No (2)

Q45 What month and year was your most recent CD4 count test? Please provide the month and year MMYYYY

________________________________________________________________

Q46 What was the result of your most recent  CD4 count test? Please select one of the answers that most closely matches your most recent CD4 count results in cells/mm3.

- Less than 50 (1)
- 51 - 200 (2)
- 201 - 350 (3)
- 351 - 500 (4)
- Greater than 500 (5)
- I don't know (6)

Q47 Are you currently taking pills or other medicines to treat your HIV (antiretroviral (ARV) medications, not medications you are taking to prevent other complications of HIV)?

- Yes (1)
- No (2)

Display This Question:

If Are you currently taking pills or other medicines to treat your HIV (antiretroviral (ARV) medicat... = Yes

Q48 What was the month and year you first started taking HIV medications? Please provide the month and year MMYYY

________________________________________________________________

Display This Question:

If Are you currently taking pills or other medicines to treat your HIV (antiretroviral (ARV) medicat... = Yes

Q49 In the last 30 days, what percent of your prescribed HIV medications have you taken? 
0% means you have taken no medication, 50% means you have taken ½ of your medication, and 100% means you have taken every single dose of your medication. If you are unsure, make your best guess.

|  | No medication | 1/2 of your medication | Every single dose of medication |
| --- | --- | --- | --- |

|  | 0 | 10 | 20 | 30 | 40 | 50 | 60 | 70 | 80 | 90 | 100 |
| --- | --- | --- | --- | --- | --- | --- | --- | --- | --- | --- | --- |

| % medication taken () | 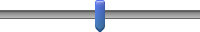 |
| --- | --- |

Display This Question:

If Are you currently taking pills or other medicines to treat your HIV (antiretroviral (ARV) medicat... = Yes

Q50 During the past 4 days, on how many days have you missed taking all your HIV medication doses?

- None (1)
- 1 day (2)
- 2 days (3)
- 3 days (4)
- 4 days (5)

Domain 3 STI Testing

Q51 A sexually transmitted infection (STI) is an infection transmitted through sexual activity, such as syphilis, gonorrhea, chlamydia, herpes, or genital warts. Have you ever been tested for an STI that was not HIV?

- Yes (1)
- No (2)
- Decline to answer (3)

| Page Break |  |
| --- | --- |

Display This Question:

If A sexually transmitted infection (STI) is an infection transmitted through sexual activity, such... = Yes

Q52 Please indicate whether you have been diagnosed with any of the following sexually transmitted infections in the past 6 months.  Please select all that apply.

- Chlamydia (1)
- Genital warts, anal warts, HPV (2)
- Gonorrhea (3)
- Hepatitis B (4)
- Hepatitis C (5)
- Herpes, HSV1/HSV2 (6)
- Syphilis (7)
- Urethritis (8)

Q53 Which of the following drugs have you used in your life? Please check all that apply.

- Tobacco (cigarettes, chewing tobacco, cigars, e-cigarettes, etc.) (1)
- Alcohol (beer, wine, spirits, etc.) (2)
- Cannabis (marijuana, pot, weed, grass, hash, synthetic cannabis, etc.) (3)
- Cocaine (coke, crack, etc.) (4)
- Amphetamines (speed, meth, diet pills, ecstasy, Ritalin, Adderall, etc.) (5)
- Inhalants (nitrous, glue, petrol, paint thinner, etc.) (6)
- Sedatives, tranquilizers, or sleeping pills (valium, Serepax, Rohypnol, Xanax, Ambien, GHB, etc.) (7)
- Hallucinogens (LSD, acid, mushrooms, PCP, Ketamine, etc.) (8)
- Opioids (heroin, morphine, methadone, codeine, Oxycotin, Percocet, Vicodin, etc.) (9)
- Decline to answer (10)

| Page Break |  |
| --- | --- |

Display This Question:

If Which of the following drugs have you used in your life? Please check all that apply. = Tobacco (cigarettes, chewing tobacco, cigars, e-cigarettes, etc.)

Q54 In the past 3 months, how often have you used tobacco products (cigarettes, chewing tobacco, cigars, e-cigarettes, etc.)?

- Never (1)
- Once or twice (2)
- Monthly (3)
- Weekly (4)
- Daily or almost daily (5)
- Decline to answer (6)

Display This Question:

If Which of the following drugs have you used in your life? Please check all that apply. = Tobacco (cigarettes, chewing tobacco, cigars, e-cigarettes, etc.)

Q55 During the past 3 months, how often have you had a desire to use tobacco products (cigarettes, chewing tobacco, cigars, e-cigarettes, etc.)?

- Never (1)
- Once or twice (2)
- Monthly (3)
- Weekly (4)
- Daily or almost daily (5)
- Decline to answer (6)

Display This Question:

If Which of the following drugs have you used in your life? Please check all that apply. = Tobacco (cigarettes, chewing tobacco, cigars, e-cigarettes, etc.)

Q56 During the past 3 months, how often has your use of tobacco products (cigarettes, chewing tobacco, cigars, e-cigarettes, etc.) led to health, social, legal or financial problems?

- Never (1)
- Once or twice (2)
- Monthly (3)
- Weekly (4)
- Daily or almost daily (5)
- Decline to answer (6)

Display This Question:

If Which of the following drugs have you used in your life? Please check all that apply. = Tobacco (cigarettes, chewing tobacco, cigars, e-cigarettes, etc.)

Q57 During the past 3 months, how often have you failed to do what was normally expected of you because of your use of tobacco products (cigarettes, chewing tobacco, cigars, e-cigarettes, etc.)?

- Never (1)
- Once or twice (2)
- Monthly (3)
- Weekly (4)
- Daily or almost daily (5)
- Decline to answer (6)

Display This Question:

If Which of the following drugs have you used in your life? Please check all that apply. = Tobacco (cigarettes, chewing tobacco, cigars, e-cigarettes, etc.)

Q58 Has a friend or relative or anyone else ever expressed concern about your use of tobacco products (cigarettes, chewing tobacco, cigars, e-cigarettes, etc.)?

- No, never (1)
- Yes, in the past 3 months (2)
- Yes, but not in the past 3 months (3)
- Decline to answer (4)

Display This Question:

If Which of the following drugs have you used in your life? Please check all that apply. = Tobacco (cigarettes, chewing tobacco, cigars, e-cigarettes, etc.)

Q59 Have you ever tried and failed to control, cut down or stop using tobacco products (cigarettes, chewing tobacco, cigars, e-cigarettes, etc.)?

- No, never (1)
- Yes, in the past 3 months (2)
- Yes, but not in the past 3 months (3)
- Decline to answer (4)

| Page Break |  |
| --- | --- |

Display This Question:

If Which of the following drugs have you used in your life? Please check all that apply. = Alcohol (beer, wine, spirits, etc.)

Q60 In the past 3 months, how often have you used alcoholic beverages (beer, wine, spirits, etc.)?

- Never (1)
- Once or twice (2)
- Monthly (3)
- Weekly (4)
- Daily or almost daily (5)
- Decline to answer (6)

| Page Break |  |
| --- | --- |

Display This Question:

If In the past 3 months, how often have you used alcoholic beverages (beer, wine, spirits, etc.)? = Once or twice

Or In the past 3 months, how often have you used alcoholic beverages (beer, wine, spirits, etc.)? = Monthly

Or In the past 3 months, how often have you used alcoholic beverages (beer, wine, spirits, etc.)? = Weekly

Or In the past 3 months, how often have you used alcoholic beverages (beer, wine, spirits, etc.)? = Daily or almost daily

Q61 How many drinks containing alcohol do you have on a typical day?

- 1 or 2 (1)
- 3 or 4 (2)
- 5 or 6 (3)
- 7 to 9 (4)
- 10 or more (5)
- Decline to answer (6)

Display This Question:

If Which of the following drugs have you used in your life? Please check all that apply. = Alcohol (beer, wine, spirits, etc.)

Q62 How often do you have 5 or more drinks on one occasion?

- Never (1)
- Less than monthly (2)
- Monthly (3)
- Weekly (4)
- Daily or almost daily (5)
- Decline to answer (6)

Display This Question:

If Which of the following drugs have you used in your life? Please check all that apply. = Alcohol (beer, wine, spirits, etc.)

Q63 During the past 3 months, how often have you had a desire to use alcoholic beverages (beer, wine, spirits, etc.)?

- Never (1)
- Once or twice (2)
- Monthly (3)
- Weekly (4)
- Daily or almost daily (5)
- Decline to answer (6)

Display This Question:

If Which of the following drugs have you used in your life? Please check all that apply. = Alcohol (beer, wine, spirits, etc.)

Q64 During the past 3 months, how often has your use of alcoholic beverages (beer, wine, spirits, etc.) led to health, social, legal or financial problems?

- Never (1)
- Once or twice (2)
- Monthly (3)
- Weekly (4)
- Daily or almost daily (5)
- Decline to answer (6)

Display This Question:

If Which of the following drugs have you used in your life? Please check all that apply. = Alcohol (beer, wine, spirits, etc.)

Q65 During the past 3 months, how often have you failed to do what was normally expected of you because of your use of alcoholic beverages (beer, wine, spirits, etc.)?

- Never (1)
- Once or twice (2)
- Monthly (3)
- Weekly (4)
- Daily or almost daily (5)
- Decline to answer (6)

Display This Question:

If Which of the following drugs have you used in your life? Please check all that apply. = Alcohol (beer, wine, spirits, etc.)

Q66 Has a friend or relative or anyone else ever expressed concern about your use of alcoholic beverages (beer, wine, spirits, etc.)?

- No, never (1)
- Yes, in the past 3 months (2)
- Yes, but not in the past 3 months (3)
- Decline to answer (4)

Display This Question:

If Which of the following drugs have you used in your life? Please check all that apply. = Alcohol (beer, wine, spirits, etc.)

Q67 Have you ever tried and failed to control, cut down or stop using alcoholic beverages (beer, wine, spirits, etc.)?

- No, never (1)
- Yes, in the past 3 months (2)
- Yes, but not in the past 3 months (3)
- Decline to answer (4)

| Page Break |  |
| --- | --- |

Display This Question:

If Which of the following drugs have you used in your life? Please check all that apply. = Cannabis (marijuana, pot, weed, grass, hash, synthetic cannabis, etc.)

Q68 Do you have or have you had a prescription to use cannabis (marijuana, pot, grass, hash, etc.) or synthetic cannabis?

- Yes (1)
- No (2)
- Decline to answer (3)

Display This Question:

If Which of the following drugs have you used in your life? Please check all that apply. = Cannabis (marijuana, pot, weed, grass, hash, synthetic cannabis, etc.)

Q69 Do you use or have you used cannabis (marijuana, pot, grass, hash, etc.) or synthetic cannabis for reasons other than prescription, or more frequently or at higher doses than prescribed?

- Yes (1)
- No (2)
- I don't know (3)

Display This Question:

If Which of the following drugs have you used in your life? Please check all that apply. = Cannabis (marijuana, pot, weed, grass, hash, synthetic cannabis, etc.)

Q70 In the past 3 months, how often have you used cannabis (marijuana, pot, grass, hash, etc.) or synthetic cannabis?

- Never (1)
- Once or twice (2)
- Monthly (3)
- Weekly (4)
- Daily or almost daily (5)
- Decline to answer (6)

| Page Break |  |
| --- | --- |

Display This Question:

If In the past 3 months, how often have you used cannabis (marijuana, pot, grass, hash, etc.) or syn... = Once or twice

Or In the past 3 months, how often have you used cannabis (marijuana, pot, grass, hash, etc.) or syn... = Monthly

Or In the past 3 months, how often have you used cannabis (marijuana, pot, grass, hash, etc.) or syn... = Weekly

Or In the past 3 months, how often have you used cannabis (marijuana, pot, grass, hash, etc.) or syn... = Daily or almost daily

Q71 During the past 3 months, how often have you had a desire to use cannabis (marijuana, pot, grass, hash, etc.) or synthetic cannabis?

- Never (1)
- Once or twice (2)
- Monthly (3)
- Weekly (4)
- Daily or almost daily (5)
- Decline to answer (6)

Display This Question:

If In the past 3 months, how often have you used cannabis (marijuana, pot, grass, hash, etc.) or syn... = Once or twice

Or In the past 3 months, how often have you used cannabis (marijuana, pot, grass, hash, etc.) or syn... = Monthly

Or In the past 3 months, how often have you used cannabis (marijuana, pot, grass, hash, etc.) or syn... = Weekly

Or In the past 3 months, how often have you used cannabis (marijuana, pot, grass, hash, etc.) or syn... = Daily or almost daily

Q72 During the past 3 months, how often has your use of cannabis (marijuana, pot, grass, hash, etc.) or synthetic cannabis led to health, social, legal or financial problems?

- Never (1)
- Once or twice (2)
- Monthly (3)
- Weekly (4)
- Daily or almost daily (5)
- Decline to answer (6)

Display This Question:

If In the past 3 months, how often have you used cannabis (marijuana, pot, grass, hash, etc.) or syn... = Once or twice

Or In the past 3 months, how often have you used cannabis (marijuana, pot, grass, hash, etc.) or syn... = Monthly

Or In the past 3 months, how often have you used cannabis (marijuana, pot, grass, hash, etc.) or syn... = Weekly

Or In the past 3 months, how often have you used cannabis (marijuana, pot, grass, hash, etc.) or syn... = Daily or almost daily

Q73 During the past 3 months, how often have you failed to do what was normally expected of you because of your use of cannabis (marijuana, pot, grass, hash, etc.) or synthetic cannabis?

- Never (1)
- Once or twice (2)
- Monthly (3)
- Weekly (4)
- Daily or almost daily (5)
- Decline to answer (6)

Display This Question:

If In the past 3 months, how often have you used cannabis (marijuana, pot, grass, hash, etc.) or syn... = Once or twice

Or In the past 3 months, how often have you used cannabis (marijuana, pot, grass, hash, etc.) or syn... = Monthly

Or In the past 3 months, how often have you used cannabis (marijuana, pot, grass, hash, etc.) or syn... = Weekly

Or In the past 3 months, how often have you used cannabis (marijuana, pot, grass, hash, etc.) or syn... = Daily or almost daily

Q74 Has a friend or relative or anyone else ever expressed concern about your use of cannabis (marijuana, pot, grass, hash, etc.) or synthetic cannabis?

- No, never (1)
- Yes, in the past 3 months (2)
- Yes, but not in the past 3 months (3)
- Decline to answer (4)

Display This Question:

If In the past 3 months, how often have you used cannabis (marijuana, pot, grass, hash, etc.) or syn... = Once or twice

Or In the past 3 months, how often have you used cannabis (marijuana, pot, grass, hash, etc.) or syn... = Monthly

Or In the past 3 months, how often have you used cannabis (marijuana, pot, grass, hash, etc.) or syn... = Weekly

Or In the past 3 months, how often have you used cannabis (marijuana, pot, grass, hash, etc.) or syn... = Daily or almost daily

Q75 Have you ever tried and failed to control, cut down or stop using cannabis (marijuana, pot, grass, hash, etc.) or synthetic cannabis?

- No, never (1)
- Yes, in the past 3 months (2)
- Yes, but not in the past 3 months (3)
- Decline to answer (4)

| Page Break |  |
| --- | --- |

Display This Question:

If Which of the following drugs have you used in your life? Please check all that apply. = Cocaine (coke, crack, etc.)

Q76 In the past 3 months, how often have you used cocaine (coke, crack, etc.)?

- Never (1)
- Once or twice (2)
- Monthly (3)
- Weekly (4)
- Daily or almost daily (5)
- Decline to answer (6)

| Page Break |  |
| --- | --- |

Display This Question:

If In the past 3 months, how often have you used cocaine (coke, crack, etc.)? = Once or twice

Or In the past 3 months, how often have you used cocaine (coke, crack, etc.)? = Monthly

Or In the past 3 months, how often have you used cocaine (coke, crack, etc.)? = Weekly

Or In the past 3 months, how often have you used cocaine (coke, crack, etc.)? = Daily or almost daily

Q77 During the past 3 months, how often have you had a desire to use cocaine (coke, crack, etc.)?

- Never (1)
- Once or twice (2)
- Monthly (3)
- Weekly (4)
- Daily or almost daily (5)
- Decline to answer (6)

Display This Question:

If In the past 3 months, how often have you used cocaine (coke, crack, etc.)? = Once or twice

Or In the past 3 months, how often have you used cocaine (coke, crack, etc.)? = Monthly

Or In the past 3 months, how often have you used cocaine (coke, crack, etc.)? = Weekly

Or In the past 3 months, how often have you used cocaine (coke, crack, etc.)? = Daily or almost daily

Q78 During the past 3 months, how often has your use of cocaine (coke, crack, etc.) led to health, social, legal or financial problems?

- Never (1)
- Once or twice (2)
- Monthly (3)
- Weekly (4)
- Daily or almost daily (5)
- Decline to answer (6)

Display This Question:

If In the past 3 months, how often have you used cocaine (coke, crack, etc.)? = Once or twice

Or In the past 3 months, how often have you used cocaine (coke, crack, etc.)? = Monthly

Or In the past 3 months, how often have you used cocaine (coke, crack, etc.)? = Weekly

Or In the past 3 months, how often have you used cocaine (coke, crack, etc.)? = Daily or almost daily

Q79 During the past 3 months, how often have you failed to do what was normally expected of you because of your use of cocaine (coke, crack, etc.)?

- Never (1)
- Once or twice (2)
- Monthly (3)
- Weekly (4)
- Daily or almost daily (5)
- Decline to answer (6)

Display This Question:

If In the past 3 months, how often have you used cocaine (coke, crack, etc.)? = Once or twice

Or In the past 3 months, how often have you used cocaine (coke, crack, etc.)? = Monthly

Or In the past 3 months, how often have you used cocaine (coke, crack, etc.)? = Weekly

Or In the past 3 months, how often have you used cocaine (coke, crack, etc.)? = Daily or almost daily

Q80 Has a friend or relative or anyone else ever expressed concern about your use of cocaine (coke, crack, etc.)?

- No, never (1)
- Yes, in the past 3 months (2)
- Yes, but not in the past 3 months (3)
- Decline to answer (4)

Display This Question:

If In the past 3 months, how often have you used cocaine (coke, crack, etc.)? = Once or twice

Or In the past 3 months, how often have you used cocaine (coke, crack, etc.)? = Monthly

Or In the past 3 months, how often have you used cocaine (coke, crack, etc.)? = Weekly

Or In the past 3 months, how often have you used cocaine (coke, crack, etc.)? = Daily or almost daily

Q81 Have you ever tried and failed to control, cut down or stop using cocaine (coke, crack, etc.)?

- No, never (1)
- Yes, in the past 3 months (2)
- Yes, but not in the past 3 months (3)
- Decline to answer (4)

| Page Break |  |
| --- | --- |

Display This Question:

If Which of the following drugs have you used in your life? Please check all that apply. = Amphetamines (speed, meth, diet pills, ecstasy, Ritalin, Adderall, etc.)

Q83 In the past 3 months, how often have you used amphetamine type stimulants (speed, meth, diet pills, ecstasy, Ritalin, Adderall, etc.)?

- Never (1)
- Once or twice (2)
- Monthly (3)
- Weekly (4)
- Daily or almost daily (5)
- Decline to answer (6)

| Page Break |  |
| --- | --- |

Display This Question:

If In the past 3 months, how often have you used amphetamine type stimulants (speed, meth, diet pill... = Once or twice

Or In the past 3 months, how often have you used amphetamine type stimulants (speed, meth, diet pill... = Monthly

Or In the past 3 months, how often have you used amphetamine type stimulants (speed, meth, diet pill... = Weekly

Or In the past 3 months, how often have you used amphetamine type stimulants (speed, meth, diet pill... = Daily or almost daily

Q84 During the past 3 months, how often have you had a desire to use amphetamine type stimulants (speed, meth, diet pills, ecstasy, Ritalin, Adderall, etc.)?

- Never (1)
- Once or twice (2)
- Monthly (3)
- Weely (4)
- Daily or almost daily (5)
- Decline to answer (6)

Display This Question:

If In the past 3 months, how often have you used amphetamine type stimulants (speed, meth, diet pill... = Once or twice

Or In the past 3 months, how often have you used amphetamine type stimulants (speed, meth, diet pill... = Monthly

Or In the past 3 months, how often have you used amphetamine type stimulants (speed, meth, diet pill... = Weekly

Or In the past 3 months, how often have you used amphetamine type stimulants (speed, meth, diet pill... = Daily or almost daily

Q85 During the past 3 months, how often has your use of amphetamine type stimulants (speed, meth, diet pills, ecstasy, Ritalin, Adderall, etc.) led to health, social, legal or financial problems?

- Never (1)
- Once or twice (2)
- Monthly (3)
- Weekly (4)
- Daily or almost daily (5)
- Decline to answer (6)

Display This Question:

If In the past 3 months, how often have you used amphetamine type stimulants (speed, meth, diet pill... = Once or twice

Or In the past 3 months, how often have you used amphetamine type stimulants (speed, meth, diet pill... = Monthly

Or In the past 3 months, how often have you used amphetamine type stimulants (speed, meth, diet pill... = Weekly

Or In the past 3 months, how often have you used amphetamine type stimulants (speed, meth, diet pill... = Daily or almost daily

Q86 During the past 3 months, how often have you failed to do what was normally expected of you because of your use of amphetamine type stimulants (speed, meth, diet pills, ecstasy, Ritalin, Adderall, etc.)?

- Never (1)
- Once or twice (2)
- Monthly (3)
- Weekly (4)
- Daily or almost daily (5)
- Decline to answer (6)

Display This Question:

If In the past 3 months, how often have you used amphetamine type stimulants (speed, meth, diet pill... = Once or twice

Or In the past 3 months, how often have you used amphetamine type stimulants (speed, meth, diet pill... = Monthly

Or In the past 3 months, how often have you used amphetamine type stimulants (speed, meth, diet pill... = Weekly

Or In the past 3 months, how often have you used amphetamine type stimulants (speed, meth, diet pill... = Daily or almost daily

Q87 Has a friend or relative or anyone else ever expressed concern about your use of amphetamine type stimulants (speed, meth, diet pills, ecstasy, Ritalin, Adderall, etc.)?

- No, never (1)
- Yes, in the past 3 months (2)
- Yes, but not in the past 3 months (3)
- Decline to answer (4)

Display This Question:

If In the past 3 months, how often have you used amphetamine type stimulants (speed, meth, diet pill... = Once or twice

Or In the past 3 months, how often have you used amphetamine type stimulants (speed, meth, diet pill... = Monthly

Or In the past 3 months, how often have you used amphetamine type stimulants (speed, meth, diet pill... = Weekly

Or In the past 3 months, how often have you used amphetamine type stimulants (speed, meth, diet pill... = Daily or almost daily

Q88 Have you ever tried and failed to control, cut down or stop using amphetamine type stimulants (speed, meth, diet pills, ecstasy, Ritalin, Adderall, etc.)?

- No, never (1)
- Yes, in the past 3 months (2)
- Yes, but not in the past 3 months (3)
- Decline to answer (4)

| Page Break |  |
| --- | --- |

Display This Question:

If Which of the following drugs have you used in your life? Please check all that apply. = Inhalants (nitrous, glue, petrol, paint thinner, etc.)

Q89 In the past 3 months, how often have you used inhalants (nitrous, glue, petrol, paint thinner, etc.)?

- Never (1)
- Once or twice (2)
- Monthly (3)
- Weekly (4)
- Daily or almost daily (5)
- Decline to answer (6)

| Page Break |  |
| --- | --- |

Display This Question:

If In the past 3 months, how often have you used inhalants (nitrous, glue, petrol, paint thinner, et... = Once or twice

Or In the past 3 months, how often have you used inhalants (nitrous, glue, petrol, paint thinner, et... = Monthly

Or In the past 3 months, how often have you used inhalants (nitrous, glue, petrol, paint thinner, et... = Weekly

Or In the past 3 months, how often have you used inhalants (nitrous, glue, petrol, paint thinner, et... = Daily or almost daily

Q90 During the past 3 months, how often have you had a desire to use inhalants (nitrous, glue, petrol, paint thinner, etc.)?

- Never (1)
- Once or twice (2)
- Monthly (3)
- Weekly (4)
- Daily or almost daily (5)
- Decline to answer (6)

Display This Question:

If In the past 3 months, how often have you used inhalants (nitrous, glue, petrol, paint thinner, et... = Once or twice

Or In the past 3 months, how often have you used inhalants (nitrous, glue, petrol, paint thinner, et... = Monthly

Or In the past 3 months, how often have you used inhalants (nitrous, glue, petrol, paint thinner, et... = Weekly

Or In the past 3 months, how often have you used inhalants (nitrous, glue, petrol, paint thinner, et... = Daily or almost daily

Q91 During the past 3 months, how often has your use of inhalants (nitrous, glue, petrol, paint thinner, etc.) led to health, social, legal or financial problems?

- Never (1)
- Once or twice (2)
- Monthly (3)
- Weekly (4)
- Daily or almost daily (5)
- Decline to answer (6)

Display This Question:

If In the past 3 months, how often have you used inhalants (nitrous, glue, petrol, paint thinner, et... = Once or twice

Or In the past 3 months, how often have you used inhalants (nitrous, glue, petrol, paint thinner, et... = Monthly

Or In the past 3 months, how often have you used inhalants (nitrous, glue, petrol, paint thinner, et... = Weekly

Or In the past 3 months, how often have you used inhalants (nitrous, glue, petrol, paint thinner, et... = Daily or almost daily

Q92 During the past 3 months, how often have you failed to do what was normally expected of you because of your use of inhalants (nitrous, glue, petrol, paint thinner, etc.)?

- Never (1)
- Once or twice (2)
- Monthly (3)
- Weekly (4)
- Daily or almost daily (5)
- Decline to answer (6)

Display This Question:

If In the past 3 months, how often have you used inhalants (nitrous, glue, petrol, paint thinner, et... = Once or twice

Or In the past 3 months, how often have you used inhalants (nitrous, glue, petrol, paint thinner, et... = Monthly

Or In the past 3 months, how often have you used inhalants (nitrous, glue, petrol, paint thinner, et... = Weekly

Or In the past 3 months, how often have you used inhalants (nitrous, glue, petrol, paint thinner, et... = Daily or almost daily

Q93 Has a friend or relative or anyone else ever expressed concern about your use of inhalants (nitrous, glue, petrol, paint thinner, etc.)?

- No, never (1)
- Yes, in the past 3 months (2)
- Yes, but not in the past 3 months (3)
- Decline to answer (4)

Display This Question:

If In the past 3 months, how often have you used inhalants (nitrous, glue, petrol, paint thinner, et... = Once or twice

Or In the past 3 months, how often have you used inhalants (nitrous, glue, petrol, paint thinner, et... = Monthly

Or In the past 3 months, how often have you used inhalants (nitrous, glue, petrol, paint thinner, et... = Weekly

Or In the past 3 months, how often have you used inhalants (nitrous, glue, petrol, paint thinner, et... = Daily or almost daily

Q94 Have you ever tried and failed to control, cut down or stop using inhalants (nitrous, glue, petrol, paint thinner, etc.)?

- No, never (1)
- Yes, in the past 3 months (2)
- Yes, but not in the past 3 months (3)
- Decline to answer (4)

| Page Break |  |
| --- | --- |

Display This Question:

If Which of the following drugs have you used in your life? Please check all that apply. = Sedatives, tranquilizers, or sleeping pills (valium, Serepax, Rohypnol, Xanax, Ambien, GHB, etc.)

Q95 In the past 3 months, how often have you used sedatives, tranquilizers, or sleeping pills (Valium, Serepax, Rohypnol, Xanax, Ambien, GHB, etc.)?

- Never (1)
- Once or twice (2)
- Monthly (3)
- Weekly (4)
- Daily or almost daily (5)
- Decline to answer (6)

| Page Break |  |
| --- | --- |

Display This Question:

If In the past 3 months, how often have you used sedatives, tranquilizers, or sleeping pills (Valium... = Once or twice

Or In the past 3 months, how often have you used sedatives, tranquilizers, or sleeping pills (Valium... = Monthly

Or In the past 3 months, how often have you used sedatives, tranquilizers, or sleeping pills (Valium... = Weekly

Or In the past 3 months, how often have you used sedatives, tranquilizers, or sleeping pills (Valium... = Daily or almost daily

Q96 During the past 3 months, how often have you had a desire to use sedatives, tranquilizers, or sleeping pills (Valium, Serepax, Rohypnol, Xanax, Ambien, GHB, etc.)?

- Never (1)
- Once or twice (2)
- Monthly (3)
- Weekly (4)
- Daily or almost daily (5)
- Decline to answer (6)

Display This Question:

If In the past 3 months, how often have you used sedatives, tranquilizers, or sleeping pills (Valium... = Once or twice

Or In the past 3 months, how often have you used sedatives, tranquilizers, or sleeping pills (Valium... = Monthly

Or In the past 3 months, how often have you used sedatives, tranquilizers, or sleeping pills (Valium... = Weekly

Or In the past 3 months, how often have you used sedatives, tranquilizers, or sleeping pills (Valium... = Daily or almost daily

Q97 During the past 3 months, how often has your use of sedatives, tranquilizers, or sleeping pills (Valium, Serepax, Rohypnol, Xanax, Ambien, GHB, etc.) led to health, social, legal or financial problems?

- Never (1)
- Once or twice (2)
- Monthly (3)
- Weekly (4)
- Daily or almost daily (5)
- Decline to answer (6)

Display This Question:

If In the past 3 months, how often have you used sedatives, tranquilizers, or sleeping pills (Valium... = Once or twice

Or In the past 3 months, how often have you used sedatives, tranquilizers, or sleeping pills (Valium... = Monthly

Or In the past 3 months, how often have you used sedatives, tranquilizers, or sleeping pills (Valium... = Weekly

Or In the past 3 months, how often have you used sedatives, tranquilizers, or sleeping pills (Valium... = Daily or almost daily

Q98 During the past 3 months, how often have you failed to do what was normally expected of you because of your use of sedatives, tranquilizers, or sleeping pills (Valium, Serepax, Rohypnol, Xanax, Ambien, GHB, etc.)?

- Never (1)
- Once or twice (2)
- Monthly (3)
- Weekly (4)
- Daily or almost daily (5)
- Decline to answer (6)

Display This Question:

If In the past 3 months, how often have you used sedatives, tranquilizers, or sleeping pills (Valium... = Once or twice

Or In the past 3 months, how often have you used sedatives, tranquilizers, or sleeping pills (Valium... = Monthly

Or In the past 3 months, how often have you used sedatives, tranquilizers, or sleeping pills (Valium... = Weekly

Or In the past 3 months, how often have you used sedatives, tranquilizers, or sleeping pills (Valium... = Daily or almost daily

Q99 Has a friend or relative or anyone else ever expressed concern about your use of sedatives, tranquilizers, or sleeping pills (Valium, Serepax, Rohypnol, Xanax, Ambien, GHB, etc.)?

- No, never (1)
- Yes, in the past 3 months (2)
- Yes, but not in the past 3 months (3)
- Decline to answer (4)

Display This Question:

If In the past 3 months, how often have you used sedatives, tranquilizers, or sleeping pills (Valium... = Once or twice

Or In the past 3 months, how often have you used sedatives, tranquilizers, or sleeping pills (Valium... = Monthly

Or In the past 3 months, how often have you used sedatives, tranquilizers, or sleeping pills (Valium... = Weekly

Or In the past 3 months, how often have you used sedatives, tranquilizers, or sleeping pills (Valium... = Daily or almost daily

Q100 Have you ever tried and failed to control, cut down or stop using sedatives, tranquilizers, or sleeping pills (Valium, Serepax, Rohypnol, Xanax, Ambien, GHB, etc.)?

- No, never (1)
- Yes, in the past 3 months (2)
- Yes, but not in the past 3 months (3)
- Decline to answer (4)

| Page Break |  |
| --- | --- |

Display This Question:

If Which of the following drugs have you used in your life? Please check all that apply. = Hallucinogens (LSD, acid, mushrooms, PCP, Ketamine, etc.)

Q101 In the past 3 months, how often have you used hallucinogens (LSD, acid, mushrooms, PCP, Ketamine, etc.)?

- Never (1)
- Once or twice (2)
- Monthly (3)
- Weekly (4)
- Daily or almost daily (5)
- Decline to answer (6)

| Page Break |  |
| --- | --- |

Display This Question:

If In the past 3 months, how often have you used hallucinogens (LSD, acid, mushrooms, PCP, Ketamine,... = Once or twice

Or In the past 3 months, how often have you used hallucinogens (LSD, acid, mushrooms, PCP, Ketamine,... = Monthly

Or In the past 3 months, how often have you used hallucinogens (LSD, acid, mushrooms, PCP, Ketamine,... = Weekly

Or In the past 3 months, how often have you used hallucinogens (LSD, acid, mushrooms, PCP, Ketamine,... = Daily or almost daily

Q102 During the past 3 months, how often have you had a desire to use hallucinogens (LSD, acid, mushrooms, PCP, Ketamine, etc.)?

- Never (1)
- Once or twice (2)
- Monthly (3)
- Weekly (4)
- Daily or almost daily (5)
- Decline to answer (6)

Display This Question:

If In the past 3 months, how often have you used hallucinogens (LSD, acid, mushrooms, PCP, Ketamine,... = Once or twice

Or In the past 3 months, how often have you used hallucinogens (LSD, acid, mushrooms, PCP, Ketamine,... = Monthly

Or In the past 3 months, how often have you used hallucinogens (LSD, acid, mushrooms, PCP, Ketamine,... = Weekly

Or In the past 3 months, how often have you used hallucinogens (LSD, acid, mushrooms, PCP, Ketamine,... = Daily or almost daily

Q103 During the past 3 months, how often has your use of hallucinogens (LSD, acid, mushrooms, PCP, Ketamine, etc.) led to health, social, legal or financial problems?

- Never (1)
- Once or twice (2)
- Monthly (3)
- Weekly (4)
- Daily or almost daily (5)
- Decline to answer (6)

Display This Question:

If In the past 3 months, how often have you used hallucinogens (LSD, acid, mushrooms, PCP, Ketamine,... = Once or twice

Or In the past 3 months, how often have you used hallucinogens (LSD, acid, mushrooms, PCP, Ketamine,... = Monthly

Or In the past 3 months, how often have you used hallucinogens (LSD, acid, mushrooms, PCP, Ketamine,... = Weekly

Or In the past 3 months, how often have you used hallucinogens (LSD, acid, mushrooms, PCP, Ketamine,... = Daily or almost daily

Q104 During the past 3 months, how often have you failed to do what was normally expected of you because of your use of hallucinogens (LSD, acid, mushrooms, PCP, Ketamine, etc.)?

- Never (1)
- Once or twice (2)
- Monthly (3)
- Weekly (4)
- Daily or almost daily (5)
- Decline to answer (6)

Display This Question:

If In the past 3 months, how often have you used hallucinogens (LSD, acid, mushrooms, PCP, Ketamine,... = Once or twice

Or In the past 3 months, how often have you used hallucinogens (LSD, acid, mushrooms, PCP, Ketamine,... = Monthly

Or In the past 3 months, how often have you used hallucinogens (LSD, acid, mushrooms, PCP, Ketamine,... = Weekly

Or In the past 3 months, how often have you used hallucinogens (LSD, acid, mushrooms, PCP, Ketamine,... = Daily or almost daily

Q105 Has a friend or relative or anyone else ever expressed concern about your use of hallucinogens (LSD, acid, mushrooms, PCP, Ketamine, etc.)?

- No, never (1)
- Yes, in the past 3 months (2)
- Yes, but not in the past 3 months (3)
- Decline to answer (4)

Display This Question:

If In the past 3 months, how often have you used hallucinogens (LSD, acid, mushrooms, PCP, Ketamine,... = Once or twice

Or In the past 3 months, how often have you used hallucinogens (LSD, acid, mushrooms, PCP, Ketamine,... = Monthly

Or In the past 3 months, how often have you used hallucinogens (LSD, acid, mushrooms, PCP, Ketamine,... = Weekly

Or In the past 3 months, how often have you used hallucinogens (LSD, acid, mushrooms, PCP, Ketamine,... = Daily or almost daily

Q106 Have you ever tried and failed to control, cut down or stop using hallucinogens (LSD, acid, mushrooms, PCP, Ketamine, etc.)?

- No, never (1)
- Yes, in the past 3 months (2)
- Yes, but not in the past 3 months (3)
- Decline to answer (4)

| Page Break |  |
| --- | --- |

Display This Question:

If Which of the following drugs have you used in your life? Please check all that apply. = Opioids (heroin, morphine, methadone, codeine, Oxycotin, Percocet, Vicodin, etc.)

Q107 In the past 3 months, how often have you used opioids (heroin, morphine, methadone, codeine, Oxycontin, Percocet, Vicodin, etc.)?

- Never (1)
- Once or twice (2)
- Monthly (3)
- Weekly (4)
- Daily or almost daily (5)
- Decline to answer (6)

| Page Break |  |
| --- | --- |

Display This Question:

If In the past 3 months, how often have you used opioids (heroin, morphine, methadone, codeine, Oxyc... = Once or twice

Or In the past 3 months, how often have you used opioids (heroin, morphine, methadone, codeine, Oxyc... = Monthly

Or In the past 3 months, how often have you used opioids (heroin, morphine, methadone, codeine, Oxyc... = Weekly

Or In the past 3 months, how often have you used opioids (heroin, morphine, methadone, codeine, Oxyc... = Daily or almost daily

Q108 In the past 3 months, has your opioid use included heroin?

- Yes (1)
- No (2)
- Decline to answer (3)

| Page Break |  |
| --- | --- |

Display This Question:

If In the past 3 months, has your opioid use included heroin? = Yes

Q109 In the past 3 months, was heroin the most common opioid that you used?

- Yes (1)
- No (2)
- Decline to answer (3)

Display This Question:

If In the past 3 months, has your opioid use included heroin? = Yes

Q110 During the past 3 months, how often have you had a desire to use opioids (heroin, morphine, methadone, codeine, Oxycontin, Percocet, Vicodin, etc.)?

- Never (1)
- Once or twice (2)
- Monthly (3)
- Weekly (4)
- Daily or almost daily (5)
- Decline to answer (6)

Display This Question:

If In the past 3 months, has your opioid use included heroin? = Yes

Q111 During the past 3 months, how often has your use of opioids (heroin, morphine, methadone, codeine, Oxycontin, Percocet, Vicodin, etc.) led to health, social, legal or financial problems?

- Never (1)
- Once or twice (2)
- Monthly (3)
- Weekly (4)
- Daily or almost daily (5)
- Decline to answer (6)

Display This Question:

If In the past 3 months, has your opioid use included heroin? = Yes

Q112 During the past 3 months, how often have you failed to do what was normally expected of you because of your use of opioids (heroin, morphine, methadone, codeine, Oxycontin, Percocet, Vicodin, etc.)?

- Never (1)
- Once or twice (2)
- Monthly (3)
- Weekly (4)
- Daily or almost daily (5)
- Decline to answer (6)

Display This Question:

If In the past 3 months, has your opioid use included heroin? = Yes

Q113 Has a friend or relative or anyone else ever expressed concern about your use of opioids (heroin, morphine, methadone, codeine, Oxycontin, Percocet, Vicodin, etc.)?

- No, never (1)
- Yes, in the past 3 months (2)
- Yes, but not in the past 3 months (3)
- Decline to answer (4)

Display This Question:

If In the past 3 months, has your opioid use included heroin? = Yes

Q114 Have you ever tried and failed to control, cut down or stop using opioids (heroin, morphine, methadone, codeine, Oxycontin, Percocet, Vicodin, etc.)?

- No, never (1)
- Yes, in the past 3 months (2)
- Yes, but not in the past 3 months (3)
- Decline to answer (4)

| Page Break |  |
| --- | --- |

Q115 Mental Health

Q116 Over the past 2 weeks, how often have you been bothered by any of the following problems?

|  | Not at all (1) | Several days (2) | More than half the days (3) | Nearly every day (4) | Decline to answer (5) |
| --- | --- | --- | --- | --- | --- |
| Little interest or pleasure in doing things (1) |  |  |  |  |  |
| Feeling down, depressed, or hopeless (2) |  |  |  |  |  |
| Trouble falling or staying asleep, or sleeping too much? (3) |  |  |  |  |  |
| Feeling tired or having little energy? (4) |  |  |  |  |  |
| Poor appetite or overeating? (5) |  |  |  |  |  |
| Feeling bad about yourself - or that you are a failure or have let yourself or your family down? (6) |  |  |  |  |  |
| Trouble concentrating on things, such as reading the newspaper or watching television? (7) |  |  |  |  |  |
| Moving or speaking so slowly that other people could have noticed, or the opposite - being so fidgety or restless that you have been moving around a lot more than usual? (8) |  |  |  |  |  |

Q117 Over the past 2 weeks, how often have you been bothered by any of the following problems?

|  | Not at all (1) | Several days (2) | More than half the days (3) | Nearly every day (4) | Decline to answer (5) |
| --- | --- | --- | --- | --- | --- |
| Feeling nervous, anxious or on edge (1) |  |  |  |  |  |
| Not being able to stop or control worrying (2) |  |  |  |  |  |
| Worrying too much about different things? (3) |  |  |  |  |  |
| Trouble relaxing? (4) |  |  |  |  |  |
| Being so restless that it is hard to sit still? (5) |  |  |  |  |  |
| Becoming easily annoyed or irritable? (6) |  |  |  |  |  |
| Feeling afraid as if something awful might happen? (7) |  |  |  |  |  |

| Page Break |  |
| --- | --- |

Q118 Sexual Behaviors 


The following questions refer to your sexual behavior with male or transfemale partners during the past 3 months. Our focus will be exclusively on anal sex. Therefore, do not include in your answers references to partners with whom you did not engage in anal sex.

Q119 In your lifetime, have you ever had anal sex? By anal sex, we mean when one person’s penis is inside their partner’s anus or rectum.

- Yes (1)
- No (2)
- Decline to answer (3)

Q120 During your life, how many males have you had anal sex with?

________________________________________________________________

Q121 In the past 3 months, how many male sexual partners have you had? 

 PLEASE NOTE: The number of partners in the past 3 months must be less than the total number of lifetime male sexual partners from the previous answer.

________________________________________________________________

Display This Question:

If If Text Response Is Equal to 1

Q122 Regarding this man:

- This man told you he was HIV negative and you had no reason to doubt it. (1)
- You knew this man was HIV positive. (2)
- You were not completely sure of this man's HIV status. (3)
- Decline to answer. (4)

Display This Question:

If If Text Response Is Greater Than 1

Q123 You said you had anal sex with ${Q121/ChoiceTextEntryValue} men in the last 3 months, Please enter numbers in the boxes below. The numbers must add up to ${Q121/ChoiceTextEntryValue}.

- Of those men, how many told you they were HIV negative and you had no reason to doubt it? (1) ________________________________________________
- Of those men, how many do you know to be HIV positive? (2) ________________________________________________
- Of those men, how many were you not completely sure of their HIV status? (3) ________________________________________________

Display This Question:

If If Text Response Is Equal to 1

Q124 In the past 3 months, did you have receptive anal intercourse with this man (you were the bottom)?

- Yes (1)
- No (2)
- Decline to answer (3)

Display This Question:

If If Text Response Is Equal to 1

Q125 About this man, did he put his penis in your rectum without a condom?

- Yes (1)
- No (2)
- Decline to answer (3)

Display This Question:

If If Text Response Is Equal to 1

Q126 In the past 3 months, did you have insertive anal intercourse with this man (you were the top)?

- Yes (1)
- No (2)
- Decline to answer (3)

Display This Question:

If If Text Response Is Equal to 1

Q127 About this man, did you put your penis in his rectum without a condom?

- Yes (1)
- No (2)
- Decline to answer (3)

Display This Question:

If If Text Response Is Equal to 1

Q128 Regarding this man, did you consider him to be...

- A man you were interested in romantically (1)
- A one-night stand or hookup (2)
- A friend with benefits (3)
- Decline to answer (4)

Display This Question:

If If Text Response Is Equal to 1

Q129 Did you disclose your HIV status to this man before you first had anal sex?

- Yes (1)
- No (2)
- Don't know (3)
- Decline to answer (4)

Display This Question:

If If Text Response Is Equal to 1

Q130 Did you disclose your HIV status to this man after you first had anal sex?

- Yes (1)
- No (2)
- Don't know (3)
- Decline to answer (4)

Display This Question:

If If Text Response Is Equal to 1

Q131 Did this man tell you he was taking PrEP (Truvada) or HIV medications?

- Yes (1)
- No (2)
- Don't know (3)
- Decline to answer (4)

Display This Question:

If If Text Response Is Greater Than 1

Q132 In the past 3 months, with how many of these men did you have receptive anal intercourse (you were the bottom)?

________________________________________________________________

Display This Question:

If If Text Response Is Greater Than 1

Q133 Of these men, how many put their penises in your rectum without a condom?

________________________________________________________________

Display This Question:

If If Text Response Is Greater Than 1

Q134 In the past 3 months, with how many of these men did you have insertive anal intercourse (you were the top)?

________________________________________________________________

Display This Question:

If If Text Response Is Greater Than 1

Q135 Of these men, how many men’s rectums did you put your penis into without a condom?

________________________________________________________________

Display This Question:

If If Text Response Is Greater Than 1

Q136 Of these men, how many were...  


Please enter numbers in the boxes below. The numbers must add up to ${Q121/ChoiceTextEntryValue}.

- A romantic interest? (1) ________________________________________________
- A one-night stand or hookup? (2) ________________________________________________
- A friend with benefits? (3) ________________________________________________

Display This Question:

If If Text Response Is Greater Than 1

Q137 To your knowledge, how many of these men were taking PrEP (Truvada) or HIV medications?

________________________________________________________________

Display This Question:

If If Text Response Is Greater Than 1

Q158 Of the ${Q123/ChoiceTextEntryValue/1} men who told you they were HIV negative and you had no reason to doubt it, how many did you disclose your HIV status to BEFORE you first had anal sex?

________________________________________________________________

Display This Question:

If If Text Response Is Greater Than 1

Q159 Of the ${Q123/ChoiceTextEntryValue/1} men who told you they were HIV negative and you had no reason to doubt it, how many did you disclose your HIV status to AFTER you first had anal sex?

________________________________________________________________

Display This Question:

If If Text Response Is Greater Than 1

Q160 Of the ${Q123/ChoiceTextEntryValue/2} men who you know to be HIV positive, how many did you disclose your HIV status to BEFORE you first had anal sex?

________________________________________________________________

Display This Question:

If If Text Response Is Greater Than 1

Q161 Of the ${Q123/ChoiceTextEntryValue/2} men who you know to be HIV positive, how many did you disclose your HIV status to AFTER you first had anal sex?

________________________________________________________________

Display This Question:

If If Text Response Is Greater Than 1

Q162 Of the ${Q123/ChoiceTextEntryValue/3} men who you were not completely sure of their HIV status, how many did you disclose your HIV status to BEFORE you first had anal sex?

________________________________________________________________

Display This Question:

If If Text Response Is Greater Than 1

Q163 Of the ${Q123/ChoiceTextEntryValue/3} men who you were not completely sure of their HIV status, how many did you disclose your HIV status to AFTER you first had anal sex?

________________________________________________________________

Q139 When was the first time you disclosed your HIV-status to a sex partner

- I have never disclosed (1)
- < 1 month (2)
- 1 - 3 months (3)
- 3 - 6 months (4)
- 6 - 12 months (5)
- > 12 months (6)

Q140 Disclosure Self-Efficacy

|  | Absolutely sure I cannot (1) | Somewhat sure I cannot (2) | Not sure (3) | Somewhat sure I can (4) | Absolutely sure I can (5) |
| --- | --- | --- | --- | --- | --- |
| I can disclose my HIV status before having sex even to a really hot new sex partner. (1) |  |  |  |  |  |
| I can disclose my HIV status before having sex even to a really hot new sex partner who I think might be HIV negative (2) |  |  |  |  |  |
| I can disclose my HIV status before having sex even if I was worried that my partner wouldn’t have sex with me if he knew (3) |  |  |  |  |  |
| I can disclose my HIV status before having sex even to a new sex partner when I'm really depressed or upset (4) |  |  |  |  |  |
| I can disclose my HIV status before having sex even to a partner who did not know I was positive the first time we had sex. (5) |  |  |  |  |  |
| I can disclose my HIV status before having sex even to a sex partner who has not told me his HIV status (6) |  |  |  |  |  |

| Page Break |  |
| --- | --- |

Q141 Disclosure Intention

|  | Strongly Disagree (1) | Disagree (2) | Neither Agree nor Disagree (3) | Agree (4) | Strongly Agree (5) |
| --- | --- | --- | --- | --- | --- |
| I plan on telling my HIV status to all of my new partners before we have sex. (1) |  |  |  |  |  |

| Page Break |  |
| --- | --- |

Q142 Disclosure: Positive Outcome Expectancies

|  | Strongly Disagree (1) | Disagree (2) | Agree (3) | Strongly Agree (4) |
| --- | --- | --- | --- | --- |
| I believe that my partner(s) will reject me if I tell him/her that I am HIV-positive (1) |  |  |  |  |
| I believe that my partner(s) will not trust me if I tell him/her that I am HIV-positive (2) |  |  |  |  |
| I fear being rejected by my sex partner(s) if I tell them that I am HIV-positive. (3) |  |  |  |  |

| Page Break |  |
| --- | --- |

Q143 Comfort with Disclosure
How comfortable are you talking about your HIV serostatus with:

|  | Very Uncomfortable (1) | Uncomfortable (2) | Neither Comfortable nor Uncomfortable (3) | Comfortable (4) | Very Comfortable (5) |
| --- | --- | --- | --- | --- | --- |
| Immediate family members (e.g. parents, siblings) (1) |  |  |  |  |  |
| Other relatives (e.g. grandparents, aunts, uncles) (2) |  |  |  |  |  |
| Sex partners (3) |  |  |  |  |  |
| Friends (4) |  |  |  |  |  |
| Health care providers (5) |  |  |  |  |  |

| Page Break |  |
| --- | --- |

Q144 Consequences of Disclosure - COSTS
When thinking about disclosure to a potential sex partner, how important are the following possible consequences?

|  | Strongly Disagree (1) | Disagree (2) | Agree (3) | Strongly Agree (4) |
| --- | --- | --- | --- | --- |
| We would fight (1) |  |  |  |  |
| Person would lecture me (2) |  |  |  |  |
| Person would blame me (3) |  |  |  |  |
| Person would be burdened (4) |  |  |  |  |
| Person would be concerned (5) |  |  |  |  |
| Relationship would get bad (6) |  |  |  |  |
| Might lose the relationship (7) |  |  |  |  |
| Person would not want to be around (8) |  |  |  |  |

Q145 Consequences of Disclosure - REWARDS
When thinking about disclosure to a potential sex partner, how important are the following possible consequences?

|  | Strongly Disagree (1) | Disagree (2) | Agree (8) | Strongly Agree (9) |
| --- | --- | --- | --- | --- |
| Keep them safe from HIV (1) |  |  |  |  |
| Understanding (2) |  |  |  |  |
| They had a right to know (3) |  |  |  |  |
| I could count on the person (4) |  |  |  |  |
| Get emotional support (5) |  |  |  |  |
| Improve the relationship (6) |  |  |  |  |
| Be more connected (7) |  |  |  |  |
| Would bring us closer (8) |  |  |  |  |
| Person could help me (9) |  |  |  |  |
| I could blow off steam (10) |  |  |  |  |

| Page Break |  |
| --- | --- |

Q146 Risk reduction communication strategies
 Have you used any of the following risk reduction communication strategies in the previous 3 months?

|  | Yes (1) | No (2) |
| --- | --- | --- |
| Told partner need to use condoms (1) |  |  |
| Refused condomless sex (2) |  |  |
| Discussed using condoms (3) |  |  |
| Agreed ahead of time on sexual risks (4) |  |  |

| Page Break |  |
| --- | --- |

Q147 Transmission Risk Beliefs

|  | Absolutely False (1) | Somewhat False (2) | Neither True nor False (3) | Somewhat True (4) | Absolutely True (5) |
| --- | --- | --- | --- | --- | --- |
| HIV can be prevented if you start taking medication right after unsafe sex (PEP) (1) |  |  |  |  |  |
| If your viral load is undetectable, there is very little chance of transmission (VIRAL LOAD) (2) |  |  |  |  |  |
| Someone on HIV treatment can infect an HIV-negative person with drug resistant strain (RESISTANCE) (3) |  |  |  |  |  |
| It is unlikely that someone on HIV treatment would transmit HIV during sex (TREATMENT) (4) |  |  |  |  |  |
| Because of PrEP, HIV-negative men do not need to be as worried about getting HIV (PrEP) (5) |  |  |  |  |  |
| Even if your viral load is undetectable, HIV can be found in your semen (SEMEN) (6) |  |  |  |  |  |

| Page Break |  |
| --- | --- |

Q156 Brief HIV Stigma Scale for Youth

|  | Strongly Disagree (1) | Disagree (2) | Neither Agree nor Disagree (8) | Agree (9) | Strongly Agree (10) |
| --- | --- | --- | --- | --- | --- |
| I have been hurt by how people reacted to learning I have HIV. (1) |  |  |  |  |  |
| I have stopped socializing with some people because of their reactions of my having HIV. (2) |  |  |  |  |  |
| I have lost friends by telling them I have HIV. (3) |  |  |  |  |  |
| I am very careful who I tell that I have HIV. (4) |  |  |  |  |  |
| I worry that people who know that I have HIV will tell others. (5) |  |  |  |  |  |
| I feel that I am not as good a person as others because I have HIV. (6) |  |  |  |  |  |
| Having HIV makes me feel unclean. (7) |  |  |  |  |  |
| Having HIV makes me feel that I’m a bad person. (8) |  |  |  |  |  |
| Most people think that a person with HIV is disgusting. (9) |  |  |  |  |  |
| Most people with HIV are rejected when others find out. (10) |  |  |  |  |  |

| Page Break |  |
| --- | --- |

Q157 Social Desirability Bias
Listed below are a number of statements concerning personal attitudes and traits. Read each item and decide whether the statement is true or false as it pertains to you personally.

|  | True (1) | False (2) |
| --- | --- | --- |
| Before voting I thoroughly investigate the qualifications of all the candidates (1) |  |  |
| I never hesitate to go out of my way to help someone in trouble. (2) |  |  |
| It is sometimes hard for me to go on with my work if I am not encouraged. (3) |  |  |
| I have never intensely disliked anyone. (4) |  |  |
| On occasion I have had doubts about my ability to succeed in life. (5) |  |  |
| I sometimes feel resentful when I don't get my way. (6) |  |  |
| I am always careful about my manner of dress. (7) |  |  |
| My table manners at home are as good as when I eat out in a restaurant. (8) |  |  |
| If I could get into a movie without paying and be sure I was not seen I would probably do it. (9) |  |  |
| On a few occasions, I have given up doing something because I thought too little of my ability. (10) |  |  |
| I like to gossip at times. (11) |  |  |
| There have been times when I felt like rebelling against people in authority even though I knew they were right. (12) |  |  |
| No matter who I'm talking to, I'm always a good listener. (13) |  |  |
| I can remember "playing sick" to get out of something. (14) |  |  |
| There have been occasions when I took advantage of someone. (15) |  |  |
| I'm always willing to admit it when I make a mistake. (16) |  |  |
| I always try to practice what I preach. (17) |  |  |
| I don't find it particularly difficult to get along with loud mouthed, obnoxious people. (18) |  |  |
| I sometimes try to get even rather than forgive and forget. (19) |  |  |
| When I don't know something I don't at all mind admitting it. (20) |  |  |
| I am always courteous, even to people who are disagreeable. (21) |  |  |
| At times I have really insisted on having things my own way. (22) |  |  |
| There have been occasions when I felt like smashing things. (23) |  |  |
| I would never think of letting someone else be punished for my wrongdoings. (24) |  |  |
| I never resent being asked to return a favor. (25) |  |  |
| I have never been irked when people expressed ideas very different from my own. (26) |  |  |
| I never make a long trip without checking the safety of my car. (27) |  |  |
| There have been times when I was quite jealous of the good fortune of others. (28) |  |  |
| I have almost never felt the urge to tell someone off. (29) |  |  |
| I am sometimes irritated by people who ask favors of me. (30) |  |  |
| I have never felt that I was punished without cause. (31) |  |  |
| I sometimes think when people have a misfortune they only got what they deserved. (32) |  |  |
| I have never deliberately said something that hurt someone's feelings. (33) |  |  |

End of Block: Default Question Block

Start of Block: Calendar Questions

Q170 What is (or will be) the date?

Month (1)

Day (2)

Year (3)

▼ January (1) ... December ~ 31 ~ 2020 (28194)

Q171 Enter a date: Qualtrics.SurveyEngine.addOnload(function () { var qid = this.questionId; var calid = qid + '_cal'; var y = QBuilder('div'); $(y).setStyle({clear:'both'}); var d = QBuilder('div',{className:'yui-skin-sam'},[ QBuilder('div', {id:calid}), y ]); var c = this.questionContainer; c = $(c).down('.QuestionText'); c.appendChild(d); var cal1 = new YAHOO.widget.Calendar(calid); cal1.render(); var input = $('QR~' + qid); $(input).setStyle({marginTop: '20px',width: '150px'}); var p =$(input).up(); var x = QBuilder('div'); $(x).setStyle({clear:'both'}); p.insert(x,{position:'before'}); cal1.selectEvent.subscribe(function(e,dates){ var date = dates[0][0]; if (date[1] < 10) date[1] = '0' + date[1]; if (date[2] < 10) date[2] = '0' + date[2]; input.value = date[1] +'-'+date[2]+'-'+date[0]; }) });

________________________________________________________________

| 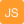 |
| --- |

Q173 Select a Date:


(Note, this question is intelligent about month lengths and leap years. You can set the year range by editing the first lines in the JS editor)

|  |  |
| --- | --- |
| Month (1) | ▼ January (1) ...   (150) |
| Day (2) | ▼ January (1) ...   (150) |
| Year (3) | ▼ January (1) ...   (150) |

End of Block: Calendar Questions
